# Unique pharmacological properties of serotoninergic G-protein coupled receptors from cestodes

**DOI:** 10.1371/journal.pntd.0006267

**Published:** 2018-02-09

**Authors:** Federico Camicia, Ana M. Celentano, Malcolm E. Johns, John D. Chan, Lucas Maldonado, Hugo Vaca, Nicolás Di Siervi, Laura Kamentezky, Ana M. Gamo, Silvia Ortega-Gutierrez, Mar Martin-Fontecha, Carlos Davio, Jonathan S. Marchant, Mara C. Rosenzvit

**Affiliations:** 1 Universidad de Buenos Aires, Consejo Nacional de Investigaciones Científicas y Técnicas, Instituto de Investigaciones en Microbiología y Parasitología Médica (IMPAM-UBA-CONICET), Facultad de Medicina, Buenos Aires, Argentina; 2 Universidad de Buenos Aires, Facultad de Medicina, Departamento de Microbiología, Parasitología e Inmunología, Paraguay, CABA, Argentina; 3 Department of Pharmacology, University of Minnesota, Minneapolis, Minnesota, United States of America; 4 Department of Biomedical Sciences, Iowa State University, Ames, Iowa, United States of America; 5 Universidad de Buenos Aires, Consejo Nacional de Investigaciones Científicas y Técnicas, Instituto de Investigaciones Farmacológicas (ININFA-UBA-CONICET), Facultad de Farmacia y Bioquímica, Buenos Aires, Argentina; 6 Departamento de Química Orgánica I, Facultad de Ciencias Químicas, Universidad Complutense de Madrid, Madrid, Spain; 7 Department of Cell Biology, Neurobiology & Anatomy; Medical College of Wisconsin; Watertown Plank Road; Milwaukee; WI; United States of America; Queen's University Belfast, UNITED KINGDOM

## Abstract

**Background:**

Cestodes are a diverse group of parasites, some of them being agents of neglected diseases. In cestodes, little is known about the functional properties of G protein coupled receptors (GPCRs) which have proved to be highly druggable targets in other organisms. Notably, serotoninergic G-protein coupled receptors (5-HT GPCRs) play major roles in key functions like movement, development and reproduction in parasites.

**Methodology/Principal findings:**

Three 5-HT GPCRs from *Echinococcus granulosus* and *Mesocestoides corti* were cloned, sequenced, bioinformatically analyzed and functionally characterized. Multiple sequence alignment with other GPCRs showed the presence of seven transmembrane segments and conserved motifs but interesting differences were also observed. Phylogenetic analysis grouped these new sequences within the 5-HT7 clade of GPCRs. Molecular modeling showed a striking resemblance in the spatial localization of key residues with their mammalian counterparts. Expression analysis using available RNAseq data showed that both *E*. *granulosus* sequences are expressed in larval and adult stages. Localization studies performed in *E*. *granulosus larvae* with a fluorescent probe produced a punctiform pattern concentrated in suckers. *E*. *granulosus* and *M*. *corti* larvae showed an increase in motility in response to serotonin. Heterologous expression revealed elevated levels of cAMP production in response to 5-HT and two of the GPCRs showed extremely high sensitivity to 5-HT (picomolar range). While each of these GPCRs was activated by 5-HT, they exhibit distinct pharmacological properties (5-HT sensitivity, differential responsiveness to ligands).

**Conclusions/Significance:**

These data provide the first functional report of GPCRs in parasitic cestodes. The serotoninergic GPCRs characterized here may represent novel druggable targets for antiparasitic intervention.

## Author summary

Cestode parasites are flatworms with the ability to parasitize almost every vertebrate species. Several of these parasites are etiological agents of neglected diseases prioritized by WHO, such as hydatid disease, or hydatidosis, a zoonosis caused by species of the genus *Echinococcus* that affects millions of people worldwide. Due to the scarcity of anthelmintic drugs available and the emergence of resistant parasites, the discovery of new anthelmintic drugs is mandatory. Neuromuscular function has been the target of commonly used drugs against parasitic diseases to impact movement, parasite development and reproduction. Here we describe three new proteins, some of them highly expressed in cestodes which could be relevant for motility. Using different approaches, the three proteins were identified as G protein coupled receptors for serotonin, an important neurotransmitter and a known modulator of cestode motility. These new receptors exhibit unique characteristics including a particular sensitivity to serotonin as well as a distinctive pharmacology, which will assist their targeting for chemotherapeutic intervention.

## Introduction

The parasitic flatworms *Echinococcus granulosus sensu lato* (s. l.) and *Mesocestoides corti* are tapeworms belonging to the class Cestoda, with *E*. *granulosus* s. l. belonging to *Taeniidae* and *M*. *corti* to *Mesocestoididae* family. The *Echinococcus* species are important parasites of wildlife, domestic animals and people worldwide. The larval stage of almost all parasites of the *E*. *granulosus* s. l. complex (which includes the species *Echinococcus granulosus sensu stricto* and *Echinococcus canadensis*) cause human cystic echinococcosis or hydatidosis, one of the 17 neglected diseases prioritized by WHO [1]. The larval stage (tetrathyridia) of *M*. *corti* has a remarkable capacity of asexual reproduction in the peritoneal cavity of mice and some other mammalian hosts [2]. This parasite is a well established model for laboratory studies and the tetrathyridium is used to examine drug effects on neuromuscular activity [3].

According to Mansour [4] the survival of parasitic helminths in their natural habitat is dependent on their ability to maintain themselves *in situ* in the face of peristaltic, blood or lymph movements. Most cestode parasites have specialized sucker-like organs to move within and attach to the host. They also exhibit well-coordinated rhythmical movements which could help to locate and maintain themselves in the host [4] or to serve the reproductive function in the parasite [5]. Any interference with coordination of the parasite movement could result in conveyance to an environment hostile for their survival or expulsion from the host [6]. These important functions can be accomplished only by the activity of different kind of muscles [7] innervated by a well-developed nervous system.

Serotonin or 5-hydroxytryptamine (5-HT) is an ancient molecule and neurotransmitter with diverse roles in organisms [8]. In invertebrates, the action of 5-HT on neuromuscular junction depends on the species and the type of preparation under consideration [9]. For example, in insects it has been shown that application of 5-HT on neuromuscular junctions appears to slightly depress synaptic strength [10]. In crustaceans, it has been demonstrated that 5-HT enhances synaptic transmission at neuromuscular junctions [11]. In the leech, 5-HT exposure has a relaxing effect on skeletal muscle but enhances muscle force and work production during locomotion and feeding [12]. Finally, in the sea cucumber, 5-HT inhibited evoked contractions induced by acetylcholine [13].

Mansour et al. [14] were among the first investigators who reported the existence of 5-HT in parasitic helminths. Work in free living planarians also highlights the diversity of serotonin receptors in flatworms [15]. Functionally, 5-HT is myoexcitatory in several species of cestodes and trematodes [3, 4, 16, 17]. However, the mechanism by which 5-HT exerts these effects remains unclear: recent work proposed the action on 5-HT receptors located on nerves [18], older reports suggest a direct effect in muscles [7], with a combination of both these effects being likely. Finally, other studies relate the motility of these worms with the activation of the glycolytic enzyme phosphofructokinase [19]. The diversity of effects is presumably enabled through the existence of multiple 5-HT receptors [20]. Evidence has accumulated that 5-HT receptors can signal through cyclic AMP (cAMP) [7, 21] and PKA [22] although other second messenger pathway**s** may also be involved [20].

The seminal idea of Mansour [17] about the potential use of 5-HT receptors as pharmacological targets in parasites has received support from recent data in planarians [23] and *Schistosoma mansoni* [18, 24, 25]. Pharmacological profiling of heterologously expressed flatworm 5-HT receptors revealed different pharmacological profiles between parasitic and human serotonin receptors and inhibitory effects of various 5-HT antagonists on motility [25]. These data strongly suggest that 5-HT receptors from parasites could be used as targets for pharmacological intervention [25].

With the exception of 5-HT_3_, which is a serotonin gated ion channel, serotonin receptors belong to the rhodopsin family or the class A of G-protein coupled receptors (GPCRs). GPCRs respond to a broad range of physicochemical entities ranging from photons, protons, calcium ions, and small molecules encompassing odorants, neurotransmitters, peptides and glycoproteins, making GPCRs versatile chemical sensors [26]. Indeed the senses of sight, smell and taste are mediated by GPCR signalling. Given the central role played by GPCRs in nearly all physiological processes, they represent attractive targets for drug discovery across a broad spectrum of diseases [26]. Serotoninergic pathways have been implicated in the aetiology of numerous disease states, including depression, schizophrenia, anxiety, social phobia, migraine, obsessive–compulsive and panic disorder [27]. Some selected examples of therapies are: clozapine, an effective agent for chronic schizophrenia [28]; the 5-HT1A receptor partial agonist buspirone, used as an anxiolytic [28]; the antipsychotic risperidone, which targets serotoninergic and dopaminergic receptors [28] and sumatriptan, a selective 5-HT1 receptor agonist used for migraine [28, 29]. Recent data demonstrate the anthelmintic praziquantel is itself a human serotoninergic ligand [30].

The availability of genome data from *Echinococcus* spp [31, 32, 33] and *M*. *corti* (http://parasite.wormbase.org/ Mesocestoides_corti_prjeb510 / Info / Index /, Helminth Genomes Consortium) has now permitted us to search for 5HT-GPCR coding genes in these cestodes.

Currently, the treatment used for echinococcosis and other cestode infections in humans relies on benzimidazoles, mainly albendazole, which is used alone or in combination with praziquantel [34, 35]. However, arising of resistance to albendazol and also to praziquantel was reported for many helminths [35, 36]. These drugs have been reported to be ineffective in 40% of cases [34], are only parasitostatics (specially for *E*. *multilocularis*), requiring of life-long treatments and are not tolerated by many patients [37]. The scarcity of anthelmintic drugs available and the emergence of resistant parasites, makes the discovery of new anthelmintic drugs an imperative need. One possible way to achieve this goal is to characterize G-protein coupled receptors in cestodes as potential pharmacological targets.

In this work, we analyzed the effect of 5-HT on motility of the tetrathyridia of *M*. *corti*. The evidence for involvement of 5-HT receptors in the cestode neuromuscular activity was reinforced by the utilization of a fluorescent probe derived from a potent agonist for the human serotoninergic receptor 5-HT_1A_ [38]. Isolation, sequencing, bioinformatic characterization and functional testing of 5-HT GPCRs were performed in two species of cestodes, with prioritized sequences modeled to identify characteristics that could be critical for receptor function. Overall, our work assesses the importance of 5-HT in the neuromuscular activity of cestodes, and identifies new 5-HT GPCRs as potential targets for drug therapy.

## Methods

### Bioinformatic analysis

Gene models coding for metabotropic 5-HT receptors were searched using the terms “5-hydroxytryptamine receptor”, “serotonin receptor”, “biogenic amine 5-HT receptor”, “G protein coupled 5 hydroxytryptamine receptor” in *Echinococcus multilocularis* [31], *Echinococcus granulosus* [32], *Echinococcus canadensis* [33] and *Mesocestoides corti* (http://parasite.wormbase.org/Mesocestoides_corti_prjeb510/Info/Index/, Helminth Genomes Consortium) databases. The retrieved gene models were also used as a query in the Blast tool from the NCBI for nucleotide (BLASTN 2.7.0+) and proteins (BLASTP 2.7.0+). The presence of conserved domains in the gene models found was determined using the Conserved Domain Database tool from the NCBI (https://www.ncbi.nlm.nih.gov/cdd/). The prediction of hypothetical open reading frames from nucleotide sequences was performed using the translate tool from the expasy proteomic tools (http://web.expasy.org/translate/). The localization and length of transmembrane domains was predicted using TMpred (http://embnet.vital-it.ch/software/TMPRED_form.html). The multiple sequence alignment was performed using the program MULTALIN [39] from PRABI (https://npsa-prabi.ibcp.fr/cgi-bin/npsa_automat.pl?page=/NPSAHLP/npsahlp_alignmultalin.html). The multiple sequence alignments were then visually inspected and manually edited when necessary. The molecular weight and isoelectric point of the predicted proteins was calculated with the program Compute pI/Mw tool (http://web.expasy.org/compute_pi/).

Cloned 5-HT_7Egran1_, 5-HT_7Egran2_ and 5-HT_7Mco1_ and predicted cestode sequences gene models ECANG7_00799 (5-HT_1Egran1_), ECANG7_02049 (5-HT_1Egran2_) and MCOS_0000684301 (5-HT_7Mco2_) were aligned with cloned serotonin receptor sequences from the following invertebrates; *Dugesia japonica* (5-HT_1Dj1_ to 5-HT_1Dj3_, 5-HT_4Dj1_ to 5-HT_4Dj5_, 5-HT_7Dj1_ to 5-HT_7Dj7_, PMCID: PMC4569474), *Schistosoma mansoni* (5-HT_7Sm_, GenBank accession number KX150867), *Caenorhabditis elegans* (UniProt IDs G5EGH0 for 5-HT_1Ce,_ O17470 for 5-HT_2Ce_ and Q22895 for 5-HT_7Ce_) and *Drosophila melanogaster* GenBank accession numbers CAA77570.1 (5-HT_1ADro_), CAA77571.1 (5-HT_1BDro_), CAA57429.1 (5-HT_2ADro_), NP_001262373.1 (5-HT_2BDro_) and NP_524599.1 (5-HT_7Dro_). Alignment of amino acid sequences was performed with Clustal Omega v1.2 [40], gaps removed with Gap Strip/Squeeze v2.1.0 (75% gap tolerance) [41], and an unrooted maximum likelihood phylogenetic tree was generated with PhyML v3.1 (500 bootstrap replicates) [42].

For molecular modelling studies, proteins studied in this work were searched using BLAST [43] against UniProtKB/Swiss-Prot databases. Protein domains were screened against PFAM, and Prosite databases using PFAM_scan [44] or HMMscan 3.0. Protein structure models were obtained using PHYRE2 [45] and SWISS-MODEL [46–49]. PDB database was used for homology searching and 3SN6 and 4IAR with ergotamine ligand (http://www.rcsb.org/pdb) [50] was used for structural comparison analyses. For all the new sequences, the template used to model the transmembrane segment of the protein was the deposited structure 3SN6, and the internal segment (intracellular loop 3) modelled using Phyre using the template 2RH1. Ramachandran Plots were produced using Rampage (http://mordred.bioc.cam.ac.uk/~rapper/rampage.php). Relevant and conserved protein residues for the ligand binding were identified and its distances from the ligand were measured. Analysis of the 5-HT_7Egran1_ modeled protein revealed that 91.7% of the residues are within the favoured regions, 4.3% in allowed regions and 4% in outlier regions of the Ramachandran plots. For the modeled 5-HT_7Egran2_ protein, 88.6% of the residues are within the favoured regions, 8.3% in allowed regions and only 3,1% in outlier regions were as for 5-HT_7Mco1_, 85.3% of the residues are within the favoured regions, 9.5% in allowed regions and 5,2% in outlier regions. All this data shows the good quality of the generated homology models (S1A, S1B and S1C Fig).

### Chemicals

Compounds UCM120 and UCM2550 were synthesized as described previously [38, 51] and dissolved in DMSO before dilution to the needed concentration. Ergotamine and tryptamine were also dissolved in DMSO whereas Lysergic acid diethylamide (LSD), tyramine, octopamine, acetylcholine, histamine and dopamine were dissolved in distilled H_2_O. 5-HT was dissolved in distilled H_2_O at stock concentration of 5 mM. Stock solutions were either made up on the day of the experiment or taken from aliquots stored at -80°C for no longer than 1 week prior to use. After 0.22 μm filtration with Millex GV filter units (Millipore, Ireland), stock solutions were diluted to the corresponding final concentration (e.g. 0.1; 1; 10; 100; 500; 1000 and 2000 μM for 5-HT) in RPMI medium with high glucose (Gibco, USA). 5-HT (H9523), LSD (L7007), ergotamine (E1200000), tryptamine (193747), tyramine (T90344), octopamine (O0250), acetylcholine (A6625), histamine (H7125) and dopamine (H8502) were obtained from Sigma Chemical Company.

### Parasite material

*Echinococcus granulosus* protoscoleces were obtained under sterile conditions by needle aspiration of hepatic hydatid cysts of porcine origin, provided by abattoirs from Buenos Aires and Santa Fe provinces, Argentina. The livers used for parasite extraction were from animals that were not specifically used for this study and all the material obtained was processed as part of the normal work of the abattoir. Samples from animals at the abattoir were collected under consent from local authorities. Protoscolex viability was assessed using the eosin exclusion test after three washes with PBS, with 50 μg/ml of gentamicin to remove cyst wall debris [52]. Only samples showing more than 95% viability were used. A fraction of the protoscoleces was used for probe imaging, other fraction for motility experiments and the remaining protoscoleces were used for species/genotype determination by sequencing a fragment of the mitochondrial cytochrome c oxidase subunit 1 (CO1), as previously described [53]. The resulting species and genotype of all protoscoleces used in this work were from *Echinococcus canadensis* G7. Three biological replicates were used with each replicate corresponding to protoscoleces obtained from a single cyst. *Mesocestoides corti* larvae (tetrathyridia) were maintained by alternate, serial passages in Wistar female rats and BALB/c female mice as previously described [54]. The tetrathyridia larvae used in drug testing were obtained after 3 months of intraperitoneal inoculation. Only tetrathyridia from up to the third serial passage in mice were used for the experiments. Three biological replicates were used with each replicate corresponding to tetrathyridia obtained from a single mouse host. Larvae freshly collected from mice were washed three times in PBS and used immediately for drug tests.

Wistar female rats and BALB/c female mice were housed at the animal facilities of Instituto de Investigaciones en Microbiología y Parasitología Médica (IMPaM), Facultad de Medicina, Universidad de Buenos Aires (UBA)-Consejo Nacional de Investigaciones Científicas y Tecnológicas (CONICET), Buenos Aires, Argentina, in a temperature-controlled light cycle room with food and water *ad libitum*.

### Ethics statement

Experiments involving the use of experimental animals were carried out according to protocols approved by the Comité Institucional para el Cuidado y Uso de Animales de Laboratorio (CICUAL), Facultad de Medicina, Universidad de Buenos Aires, Argentina (protocol “*in vivo* passages of cestode parasites from *Mesocestoides corti*” number CD N° 1127/2015). Cyst puncture was performed following the approved protocol by the same institution (protocol “Hydatid cysts puncture from natural infections” number CD N° 3723/2014).

### RNA extraction and cDNA synthesis

Total RNA from *E*. *granulosus* was extracted from protoscoleces that were crushed under liquid nitrogen and processed using Trizol reagent (Invitrogen). The RNA obtained was treated with RNase-Free DNase (Fermentas), ethanol precipitated and reverse transcribed using Superscript III reverse transcriptase (RT) (Invitrogen) and gene specific reverse primer complementary to 5-HT GPCR gene models (S1 Table). One cDNA for each selected gene model was synthesized. The same procedure was followed to obtain RNA from *M*. *corti* tetrathyridia and cDNA synthesis.

### Amplification and cloning of cDNAs coding for serotoninergic GPCRs

The gene models ECANG7_06088 and ECANG7_06092 from *E*. *granulosus* and MCOS_0000684401 from *M*. *corti*, retrieved as explained in the section “Bioinformatic analysis” were used for primer design (S1 Table) and a PCR product for each serotoninergic GPCR was obtained. The cycling parameters for ECANG7_06088 were 95°C for 5´ (initial denaturalization), then 98°C for 20´´, 56°C for 30´´ and 72°C for 2´ (repeated 5 times), then 98°C for 20´´, 66°C for 30´´ and 72°C for 2´ (repeated 35 times) and finally, 72°C for 10´ (final extension). For ECANG7_06092 were 95°C for 5´ (initial denaturalization), then 98°C for 20´´, 55°C for 30´´ and 72°C for 2´ (repeated 5 times), then 98°C for 20´´, 64°C for 30´´ and 72°C for 2´ (repeated 35 times) and finally, 72°C for 10´ (final extension). Finally, for MCOS_0000684401 were 95°C for 5´ (initial denaturalization), then 98°C for 20´´, 55°C for 30´´ and 72°C for 2´ (repeated 5 times), then 98°C for 20´´, 72°C for 30´´ and 72°C for 2´ (repeated 35 times) and finally, 72°C for 10´ (final extension). PCR reactions were performed using the KAPA HIFI polymerase (Biosystems) using the cDNA previously obtained with each reverse primer as a template. Amplification products were visualized by agarose gel electrophoresis and Gel Red staining and the bands of interest were extracted from the gel using the QIAquick Gel Extraction Kit (Qiagen), used for a non-templated adenine adding or A-tailing procedure employing a nonproofreading DNA polymerase (Pegasus, Embiotec) and finally cloned into the TOPO TA Vector (Invitrogen). The recombinant plasmids were used for *Escherichia coli* (DH5α) transformation and the transformed bacteria were grown in LB with ampicillin and kanamycin.

The selected colonies were then used for plasmid purification using the GeneJet Plasmid miniprep kit (Fermentas) and sequencing using an Applied Biosystems Big Dye terminator kit (Applied Biosystems) on an ABI 377 automated DNA sequencer. The cloned cDNA products obtained with *E*. *granulosus* primers were designed from the ECANG7_06088 and ECANG7_06092 gene models and named 5-HT_7Egran1_ and 5-HT_7Egran2_ respectively (S1 Text) following the naming scheme proposed by Tierney [55]. The cDNA cloned from the gene model MCOS_0000684401 from *M*. *corti* was named 5-HT_7Mco1_ (S1 Text).

### Motility index measurement

For the motility index measurement, the method of Camicia et al. [16] was followed with minor modifications. In the case of *E*. *granulosus*, protoscoleces were mantained in RPMI medium with high glucose (Gibco, USA) with 50 μg/ml of gentamicin for no more than 48 hours in order to avoid the potential differentiation of the protoscolex towards premicrocyst [16]. The day before the experiment the protoscoleces were transferred to U-shape 96-well microplates (Greiner Bio-One, Germany) with approximately 125 parasites per 100 μl per well. This number of parasites per well was empirically determined as the best number of worms to perform the experiments. Movement was measured as described for *C*. *elegans* [56] using a worm tracker device (WMicrotracker Designplus SRL, Argentina) which determines the motility index by the light-microbeam (100 μm wide; k = 880 nm; intensity <1 mW) scattering produced by the movement of parasites. Quantification of the signal was performed by applying a mathematical algorithm previously described for sperm viability determination (Patent number: US4176953, year of submission: 1978, year of publication: 1979).

To study the effect of 5-HT on the motility of *M*. *corti* tetrathyridia, only one worm per well was used as this was the empirically determined optimal number. Addition of more tetrathyridia to each well plate did not result in a better signal. *M*. *corti* tetrathyridia were used fresh, immediately after the mice was opened. One tetrathyridium per well in 100 μl of RPMI with high glucose (Gibco) was pipetted to U-shape 96-well microplates and incubated overnight at 37°C in a 5% CO_2_ atmosphere. The following day, basal activity recordings were performed for 2 h at 37°C in the worm tracker. After the initial 2 h, 25 μl of the same medium containing 5-HT at the indicated concentrations were added and the motility of parasites was recorded in the resultant 125 μl of medium for another 2 hours. Twenty-five microliters of fresh medium alone were added to the controls before registering motility in the resultant 125 μl. In order to avoid any potential difference between individuals not related to the effect of the 5-HT, the activity found in each well was divided by the activity found in the same well in the basal record. Each experiment was performed using 16 technical replicates per treatment condition. In order to count with biological replicates, four independent experiments, with tetrathyridia coming from different mice were performed.

### Effect of the 5-HT GPCR agonist UCM2550 on cestode larval motility

We evaluated the effect of UCM2550, a 5-HT_1A_ agonist in mammals and the parent ligand of UCM120, [38, 57] on protoscolex movement. Protoscoleces were incubated in 96-well microplates as described in the section “Motility index measurement”, and basal activity was registered for 2 hours at 37°C. After this first acquisition they were subjected, in groups of 16 replicates, to the inidicated concentrations of the previously mentioned drug, and recorded for an additional 2 hours in the resultant 125 μl. Control wells were incubated in 125 μl of medium with or without 2% of DMSO (the vehicle used to dissolve the drugs) and no differences were observed between those groups. Three independent experiments, with protoscoleces from different hydatid cysts (biological replicates), were performed. The effect of the drug on protoscolex viability was tested for toxicity at the maximal concentrations used.

### Measurement of motor activity by video imaging

In some experiments, alongside to the worm tracker motility analyses, the parasite motor activity was also measured with a digital video camera (Kodak easy share Z915 digital camera) coupled to an inverted microscope (Nikon, model TMS-F). Fresh parasites were processed in the same way as in previous sections in U-shape 96-well microplates, then incubated overnight at 37°C in a 5% CO_2_ atmosphere and finally video recorded with a digital camera after drug treatment. Images were obtained for a period of 15 seconds and the data were analyzed with ImageJ software (version 1.48, NIH, USA). Protoscoleces showed complex motor movements characterized by sucker and rostellum movements and repeated body bends. To quantify this type of movements the method of Patocka et al. [18] was followed with some modifications. The videos were transformed to stacks of TIFF images by using the Filmora video editor (Wondershare Filmora, version 8.2.3, downloaded from https://filmora.wondershare.es/) and then imported into ImageJ. First, the stacks were first converted to 8 bit type images. Next, the background was subtracted and then converted to a binary image by threshold adjust. Then, the maximum intensity of Z projection (which shows all the elements in the movie) was calculated together with the minimum intensity projection (which shows only the constant elements in the movie). Then subtracting the minimal from maximal projections with the “image calculator” it was possible to remove everything constant in the movie, including immobile animals. Finally, the resultant pixels were quantified using the “Analyze particles” command and the integrated density in the summary result table showed the pixels that changed during the course of each movie. This procedure was repeated for each video and a graphic representation of pixel change was done for each treatment (S2 Fig). Eight technical replicates and three independent experiments, with protoscoleces coming from different hydatid cysts (biological replicates), were performed.

### Whole mount probe imaging

Protoscoleces were fixed for 4 hours at room temperature (RT) in 4% (w/v) paraformaldehyde (PFA) in PBS (pH 7.4) and washed for 24 hours at RT in PBS containing 0.35% (v/v) triton X-100 (TX-100), 0.1% (w/v) sodium azide (NaN_3_), 0.1% (w/v) BSA (Sigma), 6.25 x 10^−3^ (w/v) digitonin (Dig) and 0.5% DMSO (PBS/TX-100/NaN_3_/BSA/Dig/DMSO). Approximately, 10 μl of pellet of protoscoleces were aliquoted in eppendorf tubes and incubated for 120 hours (five days) at 4° C in the presence of 100 μM of the probe UCM120 (which is a 5-HT_1A_ agonist in mammals labeled with a dansyl group as fluorescent tag) [38] in constant agitation in a final volume of 300 μl. Specimens were then washed three times with PBS and fixed for 3 hours at room temperature (RT) in 4% (w/v) paraformaldehyde (PFA) in PBS/TX-100/NaN_3_/BSA/Dig/DMSO. The protoscoleces were washed again, mounted in glycerol/PBS (8:1 v/v) and then viewed under standard fluorescence microscope (Nikon Eclipse E600) or in a confocal scanning laser microscope (CSLM, Fluoview 1000 Olympus). The individual pictures obtained from the optical sections were used for the reconstruction of the whole specimen using the program ImageJ. In order to determine if the signal obtained was specific, several controls were run in parallel: protoscoleces without probe or protoscoleces in the presence of 1000 μM of serotonin as negative control. This experiment was performed several times with similar results.

### Levels of GPCR expression

The transcriptional expression levels (in RPKM, or reads per kilobase per million reads) for each serotoninergic receptor in *Echinococcus granulosus sensu stricto* (G1 genotype) were from Zheng et al. ([32], Supplementary Table 28).

### Cell culture and cAMP assays

HEK-293 cells (ATCC CRL-1573.3) were cultured in growth media [DMEM (Gibco), 10% heat inactivated fetal bovine serum (Gibco), penicillin (100units/mL), streptomycin (100μg/mL) and L-glutamine (290μg/mL)] and used for assays between passages 5 and 25. For cestode GPCR heterologous expression assays, cells were transfected (Lipofectamine 2000, Invitrogen) at 80% confluency approximately 16 hours after seeding within T-25 culture flasks with a 1:1 ratio of human codon optimized cestode GPCR cDNA (subcloned into a pcDNA3.1(-) mammalian expression vector) and cDNA encoding the pGloSensor 22-F plasmid (Promega). The following day, cells were trypsinized, centrifuged (300g/5min), resuspended in DMEM supplemented with 1% dialyzed FBS (Gibco) and plated in 96 well, solid white plates (Corning, cat # 3917). After overnight culture to allow adherence, media was exchanged for assay buffer (HBSS supplemented with 0.1% BSA, 20mM HEPES (pH 7.4), and GloSensor reagent (Promega). cAMP-luminescence assays were performed following addition of a phosphodiesterase inhibitor (3-isobutyl-1-methylxanthine, IBMX; 200μM) using a GloMax-Multi Detection System plate reader (Promega) as described previously [24].

### Statistical analyses

Statistical analyses were carried out using the GraphPad Prism Software package, version 6 for Windows (GraphPad Software, CA, USA). To analyze the effects of serotonin in motility assays performed with the WMicrotracker device, the relative motility indices of all replicate wells for each concentration of the different independent experiments were made into one cohort for one-way ANOVA. To determine significant differences a Bonferroni and Dunnett post-tests were performed comparing all concentrations with the control. In the experiment evaluating the effects of UCM2550 on protoscolex motility, the mean motility index obtained with the WMicrotracker was compared before and after the addition of the drug with a Student’s t-test. For image based assays, pixel changes in control and treatments were calculated and analyzed with one way ANOVA and Dunnett post-test were performed comparing all the concentrations with the control. Differences from control with P < 0.05 were considered statistically significant inductions or inhibitions of motor activity.

## Results

### Cloning, sequencing and bioinformatic analysis of serotoninergic GPCRs in two cestode species

**Fig 1:**
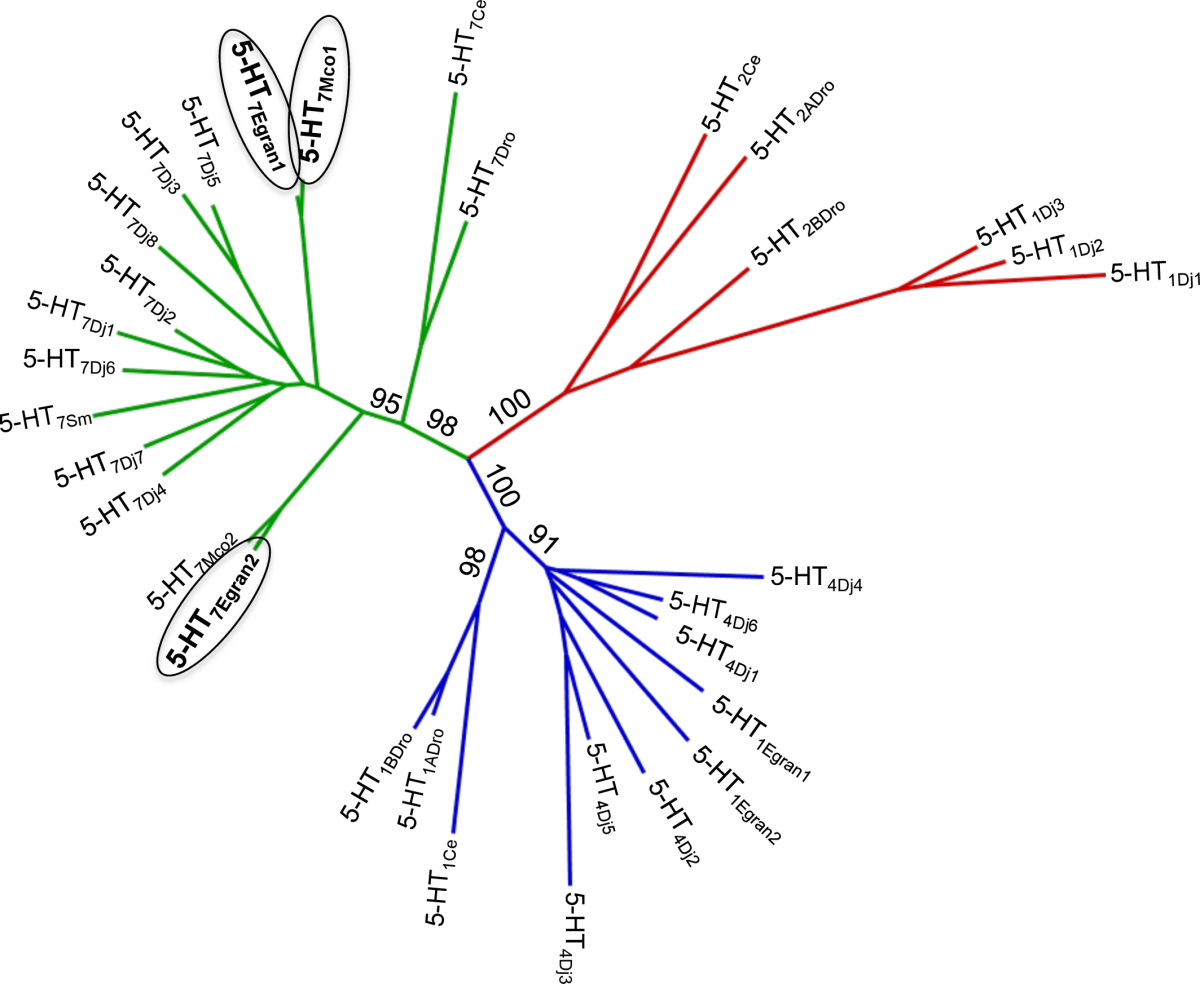
Cladogram of cestode and invertebrate serotoninergic GPCRs. The amino acid sequences of predicted cestode serotonin receptors were aligned with the repertoire of serotonin receptors cloned from various other flatworm and non-flatworm invertebrate model organisms; *Dugesia japonica*, 5-HT_Dj_; *Schistosoma mansoni*, 5-HT_Sm_; *Caenorhabditis elegans*, 5-HT_Ce_; *Drosophila melanogaster*, 5-HT_Dm_. The cestode GPCR sequences cloned and functionally expressed in this study (5-HT_7Egran1_, 5-HT_7Egran2_, 5-HT_7Mco1_) cluster within a clade of 5-HT7 like receptors (green), as does an additional predicted *M*. *corti* sequence (5-HT_7Mco2_). Other predicted cestode serotoninergic sequences (5-HT_1Egran1_ and 5-HT_1Egran2_) cluster within a clade of 5-HT1 like receptors (blue). See methods for complete list of UniProt and GenBank sequence identifiers used in this analysis. Analysis was bootstrapped with 500 replicates.

Six gene models in total were retrieved by blast searches from which four gene models were from *Echinococcus spp*. databases and only two from the *M*. *corti* database. From all them, only ECANG7_06088, ECANG7_06092 (*E*. *granulosus*) and MCOS_0000684401 (*M*. *corti*), were used for primer design since the rest of the gene models were either incomplete (lacking one or more transmembrane segments), or missing important residues for function, or they could not be amplified in PCR reactions. By using sequence specific primers, three cDNAs were cloned by standard RT-PCR being two of the sequences obtained from *E*. *granulosus* (5-HT_7Egran1_ and 5-HT_7Egran2_) and one from *M*. *corti* (5-HT_7Mco1_). The 5-HT_7Egran1_ cDNA has 2100 bp and encodes a protein of 659 amino acids with a predicted molecular weight of 73.8 kDa and a pI close to 10 (pI = 9.83). The second cDNA cloned, 5-HT_7Egran2_, has 1731 bp and encodes for a protein of 576 amino acids with a predicted molecular weight of 65 kDa and a pI close to 9 (pI = 9.12). Finally, the *M*. *corti* cDNA 5-HT_7Mco1_, has 2091 bp and encodes for a protein of 696 amino acids with a predicted molecular weight of 78.6 kDa and a pI of 9.45. Domain searches showed that all the cloned sequences have best hits with the 5-HT_7_ domain of serotonin receptors. S3 Fig shows a multiple sequence alignment between the *Homo sapiens*, *C*. *elegans*, *S*. *mansoni*, *E*. *granulosus* and *M*. *corti* sequences encoding for serotoninergic G-protein coupled receptors. The bioinformatic analysis of the cloned sequences shows several characteristics of the molecular signature of a GPCR. For example, all three sequences have seven conserved hydrophobic transmembrane segments consistent with GPCR architecture (S3 Fig). Topological modeling (TMpred) predicts an extracellular N-terminus and an intracellular C-terminus. Intracellular loop 3 and, for 5HT_7Egran1_ and 5HT_7Mco1_ the carboxy-terminal regions, were longer than the corresponding regions in the human counterpart (S3 Fig). The multiple sequence alignment also highlights conserved amino acid residues that are invariant within the rhodopsin family of GPCRs [58]. The first of them is the DRY motif present in the third transmembrane domain probably involved in the stabilization of the ground state [59, 60]. The second relevant motif found was the NPxxY motif located toward the cytoplasmic end of the seventh transmembrane domain and potentially implicated as crucial in the recruitment of β-arrestin, which is a mechanism of receptor inactivation [59–61]. The third is the PIF motif, which is a critical trigger motif for receptor activation [59, 60]. And the last one, in the transmembrane segment 6, there is a cluster of aromatic residues referred as “toggle switch” in which it was reported that the movement of some of these residues could be important for receptor activation [60]. Taking the residue position from the human 5HT_1B_ receptor as a reference sequence [62] and the Ballesteros and Weinstein nomenclature [63], the residues conserved in all the cestode sequences were: C122^3.25^ which makes putative disulphide bond contact with the C199^El2^ in the extracellular loop 2; D129^3.32^, which forms a salt bridge with the positively charged amino group of ergotamine in the human receptor [62]; C133^3.36^, which recognizes the amine portion of the ligand; T134^3.37^, which forms hydrogen bond with the indole group; A213^5.46^, which interacts with the indole ring in the ligand; W327^6.48^, F330^6.51^ and F331^6.52^, which form the orthosteric binding pocket. The residue Y359^7.43^ is hypothesized to form hydrogen bond with N6 of ergotamine in the human receptor. The residue conserved in all but not in 5-HT_7Egran2_, is T216^5.43^, which interacts with the indole ring in the ligand and is replaced by C in this position in the 5-HT_7Egran2_ sequence. The conserved S215^5.42^ of TM5 is replaced by alanine in all the cestode sequences and, interestingly, the same substitution was observed in the S7.1 receptor from the planaria *D*. *japonica* [15] and also in the Sm5HTR from *S*. *mansoni* [18]. TM5 residues (S4 Fig) are important players in ligand recognition [62, 64]: with the exception of the alanine at position 5.42, the other crucial residues (5.43 and 5.46) are conserved in mammalian serotonin receptors, particularly in subtypes 1 (5-HT1) and 7 (5-HT7). Besides ligand binding, other residues potentially involved in G protein contacts could be identified (S3 Fig). For example, the residue L316^6.37^ in the cytoplasmic end of TM6 upon activation contacts a universally conserved leucine of the G protein in the β2 adrenergic receptor-Gs structure [65]. Moreover, in the V155 position of the human 5-HT1 receptor (F139 in the human beta 2 adrenergic receptor), located in the IL2 helix, it was reported a F (phenylalanine) or L (leucine) for Gs coupling which is coincident with the residues observed in serotonin receptors from cestodes at this position [66] (S3 Fig). The phylogenetic analysis (Fig 1) shows that all the cloned sequences grouped with the 5HT_7_ subtype (S7-like clade) of invertebrate serotoninergic G-protein coupled receptors, which are Gs coupled [67].

### The transcriptomic data reveals a highly expressed receptor at the protoscolex stage

Transcriptomic data obtained from Zheng and coworkers [32], reveals that two of the receptors cloned here (5-HT_7Egran1_ and 5-HT_7Egran2_) are highly expressed in the activated protoscolex stage (S5 Fig) with the levels of expression of 5-HT_7Egran2_ being particularly high. The levels of expression of both receptors in activated protoscolex stage are at least fourfold higher than those observed in other stages of the parasite. No expression of these two receptors was detected in the oncosphere.

### Molecular modelling of 5-HT_7Egran1_, 5-HT_7Egran2_ and 5-HT_7Mco1_

**Fig 2:**
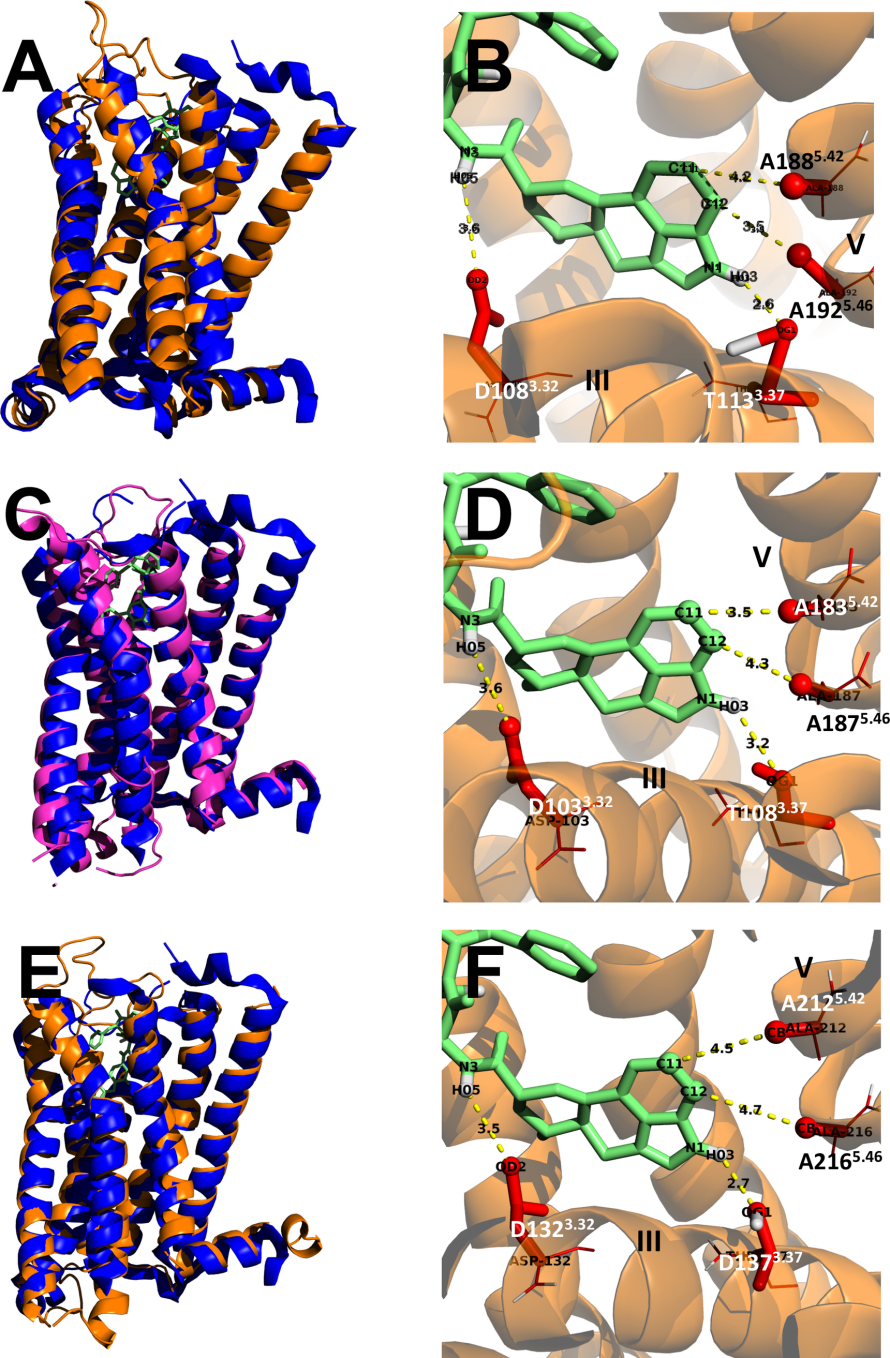
Molecular modeling of serotoninergic receptors from cestodes. The structural similarities between three cestode receptors and the published structure of the serotoninergic receptor from human (4IAR) are shown. A) Model of the serotonin receptor 5-HT_7Egran1_ (Brown) superposed to the published structure of the human serotonin receptor 5-HT1B (blue). B) Close view of the putative ergotamine interaction with residues of the transmembrane domains III and V from 5-HT_7Egran1_ (lateral chains of the amino acids involved are indicated in red), molecular distances between atoms are indicated. C) Model of 5-HT_7Egran2_ (violet) superposed to 5-HT1B (blue). D) Close view of the ergotamine interaction with some residues of the transmembrane domains III and V from 5-HT7_Egran2_. E) Model of 5-HT_7Mco1_ (Brown) superposed to 5-HT1B (blue). F) Close view of the ergotamine interaction with some residues of the transmembrane domains III and V from 5-HT_7Mco1_. In all the representations, the molecule of ergotamine was marked in green and the residues in transmembrane domains potentially involved in ergotamine interaction were marked in red.

Molecular models of 5-HT_7Egran1_, 5-HT_7Egran2_ and 5-HT_7Mco1_ were developed to explore potential structural similarities and differences between the parasitic and human receptors. Fig 2 shows the models of each cloned serotoninergic GPCR compared with the crystal structure of human 5-HT_1B_R (4IAR) [62]. A striking structural similarity in the transmembrane domains can be observed (Fig 2, models A, C and E). According to the models obtained, four important interactions with ergotamine could occur at the transmembrane segments three (III) and five (V), (Fig 2, models B, D and F). The indicated residues comprise part of the orthosteric binding pocket of the cestode GPCRs.

### Serotonin increases the motility of *Mesocestoides corti* tetrathyridium

Previously, we have shown that 5-HT induces *E*. *granulosus* protoscolex motility [16]. In spite of some evidence about 5-HT action on *M*. *corti* motility [3], the kinetics of this important neurotransmitter in motility over longer periods of time (>30 minutes) was unknown. For this reason, we analyzed further the effect of 5-HT on the larvae of *M*. *corti*. The addition of serotonin to tetrathyridia resulted in the stimulation of the motility above basal levels (S6 Fig, S1 Video). The channel activity traces show a stimulation in frequency of movement at high doses of 5-HT compared to the basal condition (S6A Fig). The dose-response curve showed a significant rise of motility starting from 100 μM of 5-HT (*p< 0.05) until the highest concentration tested of 2000 μM over a period of two hours (****p< 0.001; S6B Fig). Dose-response curves were also collected over different intervals of time (30, 60, 90 and 120 minutes). These graphs showed that the shape of the curve changes with time (S6C Fig). At the highest concentrations tested (500 to 2000 μM), the relative motility tends to drop with time after the first 30 minutes of incubation, losing their significance respect to control, probably reflecting an inactivation process. At the lowest dose (100 μM) the motility tends to rise during the first 90 minutes of incubation and then also it tends to fall after 120 minutes of incubation (S6C Fig). Similar concentrations of 5-HT (1000 μM; ***p< 0.001) are necessary to see significant changes in motility over a period of 30 minutes by video imaging (S6D Fig).

### Functional expression and pharmacological profiling show activation by 5-HT with distinct pharmacological properties

**Fig 3:**
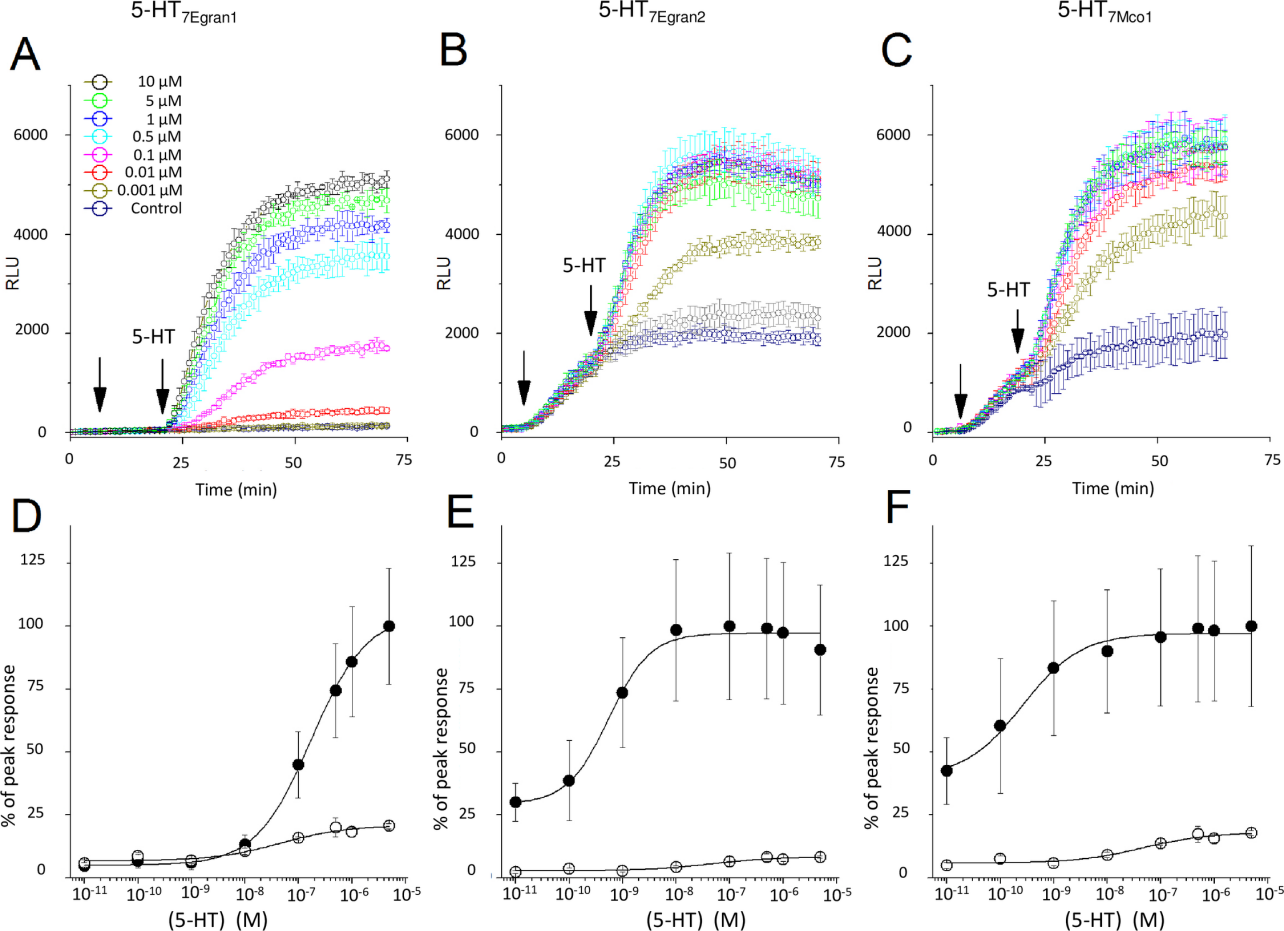
Heterologous expression of GPCRs reveals 5-HT evoked cAMP accumulation. Time resolved measurements of cAMP accumulation in cells expressing individual cestode serotoninergic GPCRs (A) 5-HT_7Egran1_, (B) 5-HT_7Egran2_ and (C) 5-HT_7Mco1_ before and after addition of IBMX (1^st^ arrow, 200μM) and different doses of 5-HT (2^nd^ arrow, doses indicated in legend). (D, E & F) dose response relationships to 5-HT measuring peak amplitude of 5-HT evoked luminescence change in cells expressing the indicated GPCR (solid circles), or untransfected HEK-293 cells (open circles).

**Fig 4:**
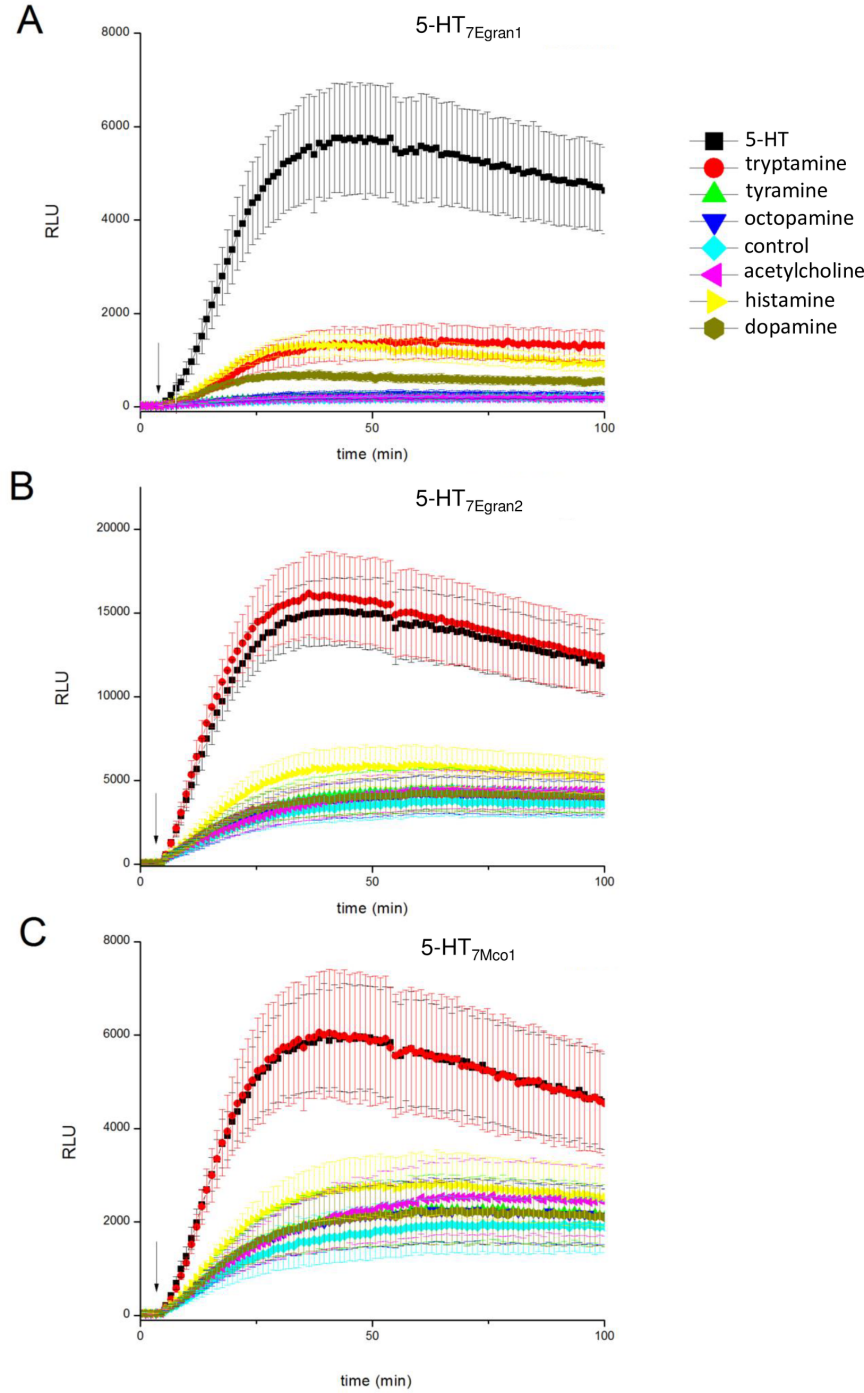
Profiling neurotransmitter specificity against cestode serotoninergic GPCRs. Responses (raw luminescence units, RLU) in cells transfected with (A) 5-HT_7Egran1_, (B) 5-HT_7Egran2_ and (C) 5-HT_7Mco1_ to indicated neurotransmitters at the indicated time points in the arrow (10μM), with the exception of 5-HT (700nM in (A), 3nM in (B) and 2nM in (C)).

Based on the bioinformatic prediction that these sequences encode serotoninergic GPCRs, responses to 5-HT were compared between cells expressing individual cestode GPCRs and untransfected cells. For each of the clones, addition of 5-HT in transfected cells evoked a rapid and dose dependent stimulation of cAMP accumulation (Fig 3A, 3B & 3C). One notable feature of two of the GPCRs– 5-HT_7Egran2_ and 5-HT_7Mco1_ –was resolution of a background rate of cAMP accumulation immediately on IBMX addition prior to addition of 5-HT (the period between the arrows in Fig 3B & 3C). This was not observed with the 5-HT_7Egran1_ clone (Fig 3A). Because of this basal coupling, the fold-change in luminescence signal on addition of 5-HT was lower with– 5-HT_7Egran2_ (~3.3-fold) and 5-HT_7Mco1_ (~2.5-fold) than observed in cells expressing 5-HT_7Egran1_ (~20-fold). Full dose response relationships were performed for each GPCR, and compared with endogenous responses to the same concentrations of 5-HT in untransfected cells (Fig 3D, 3E & 3F). The EC_50_ for 5-HT evoked cAMP generation was 171±57nM in cells expressing 5-HT_7Egran1_. For the two GPCRS exhibiting basal coupling, the EC_50_ for 5-HT evoked cAMP production was in the picomolar range measured as 543±107pM for 5-HT_7Egran2_ and 255±63pM for 5-HT_7Mco1_. Responses to 5-HT were considerably smaller in untransfected cells (Fig 3D, 3E & 3F). To investigate the specificity of these GPCRs for 5-HT, responses to higher concentrations (10μM) of other neurotransmitters (tryptamine, tyramine, octopamine, histamine, dopamine and acetylcholine) were examined (Fig 4). In all cases, 5-HT elicited maximal cAMP accumulation. At higher doses (10 μM), tryptamine also proved an activator of 5-HT_7Egran2_ (EC_50_ ~0.8μM) and 5-HT_7Mco1_ (EC_50_ ~1μM) but not 5-HT_7Egran1_ (Fig 4). The first 2 receptors were at least 1000-fold less sensitive to tryptamine than to 5-HT. Responses to other ligands were considerably smaller, likely representing responses from endogenous GPCRs in HEK-293 cells (histamine, dopamine) or comparable with levels of luminescence seen with vehicle control. From this dataset we conclude that each of the cestode sequences encodes a *bona fide* serotoninergic GPCR.

**Fig 5:**
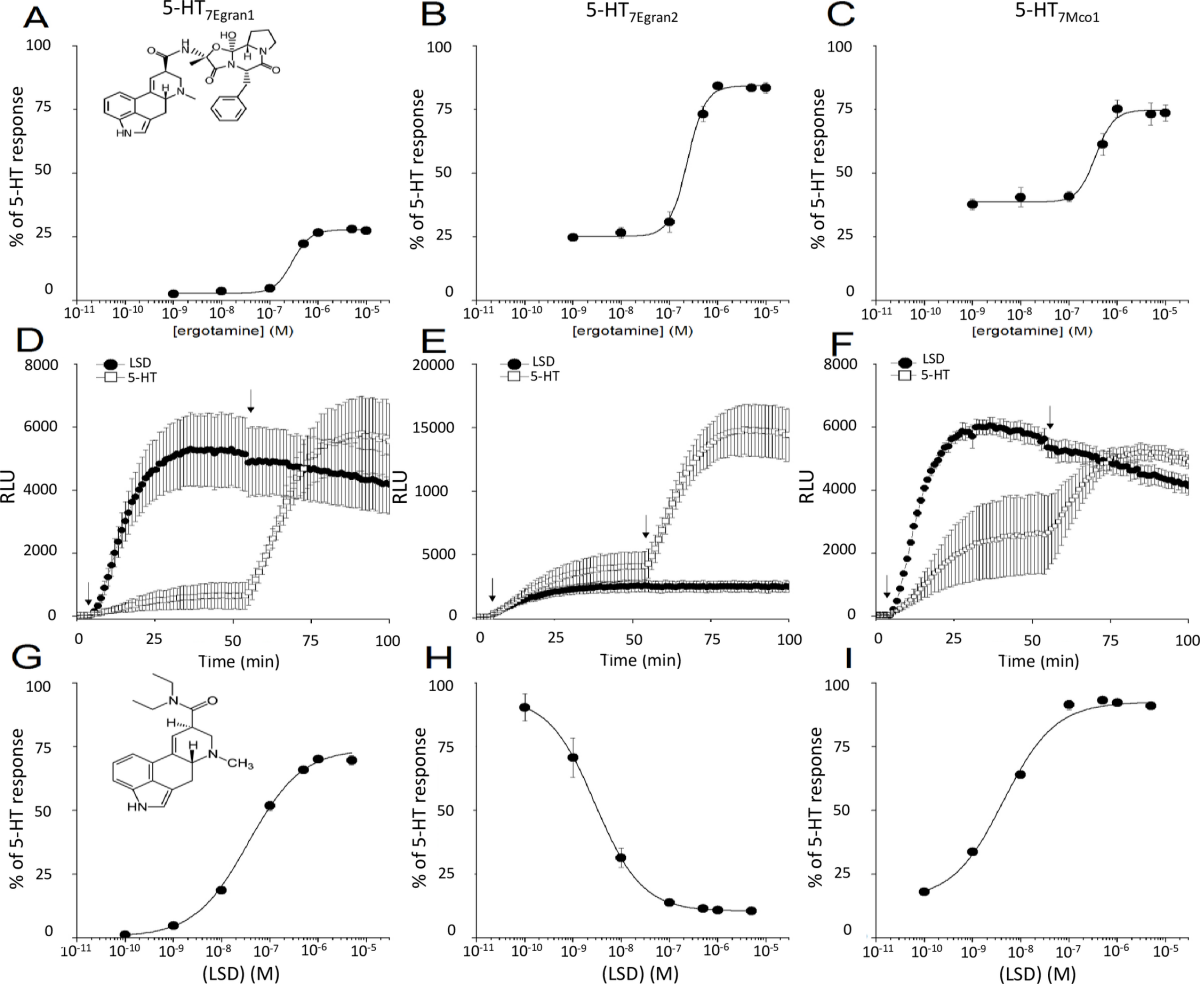
Ergot alkaloid activity at cestode 5-HT GPCRs. (A-C) Dose response relationships describing sensitivity and magnitude of peak cAMP accumulation versus maximal 5-HT response to various doses of ergotamine at (A) 5-HT_7Egran1_, (B) 5-HT_7Egran2_ and (C) 5-HT_7Mco1_ (D-F). Real time kinetic profiling of cAMP accumulation in response to an addition of either LSD and IBMX (10μM and 200μM, respectively, closed circles) or IBMX alone (200μM, open circles) at first arrow and then subsequent addition of 5-HT (5 μM, second arrow) for (D) 5-HT_7Egran1_ or 5-HT (1 μM, second arrow) for (E) 5-HT_7Egran2_ and (F) 5-HT_7Mco1_. Dose response relationships for activation or blockade of 5-HT evoked cAMP accumulation by LSD at (G) 5-HT_7Egran1_, (H) 5-HT_7Egran2_ and (I) 5-HT_7Mco1_.

Our modelling studies supported an interaction of the ergot alkaloid ergotamine with the binding pocket of the cestode GPCRs (Fig 2B, 2D & 2F). Therefore we assayed responsiveness of each GPCR to ergotamine, as well as the synthetic hallucinogen lysergic acid diethylamide (LSD) which has proved a useful probe ligand for studying 5-HT receptor properties [59]. Ergotamine activated each of the cestode 5HT_7_Rs but with variable efficacy (Fig 5A, 5B & 5C), behaving as a low efficacy partial agonist at 5-HT_7Egran1_ and a higher efficacy partial agonist at 5-HT_7Egran2_ and 5-HT_7Mco1_. Each GPCR responded to ergotamine over a similar concentration range (EC_50_s of 290±49nM, 234±37nM and 354±62nM for 5-HT_7Egran1_, 5-HT_7Egran2_ and 5-HT_7Mco1_ respectively, Fig 5A, 5B & 5C). Responses to LSD were then examined. Two of the receptors (5-HT_7Egran1_ and 5-HT_7Mco1_) were potently activated by addition of LSD but no response was observed in cells expressing 5-HT_7Egran2_ when probed in ‘agonist’ mode (Fig 5D, 5E & 5F). Rather, LSD attenuated the basal coupling of 5-HT_7Egran2_ and blocked cAMP accumulation in response to a subsequent addition of 5-HT (‘antagonist mode’), demonstrating LSD acted as an antagonist of 5-HT_7Egran2_. Full dose response relationships to LSD were performed (Fig 5G, 5H & 5I) which demonstrated EC_50_s for activation of 35±6nM (5-HT_7Egran1_) and 4.4±1.0nM (5-HT_7Mco1_), and an IC_50_ for inhibition of 5-HT responses of 2.9±0.2nM (5-HT_7Egran2_). Collectively, these functional expression data demonstrate that while each of these GPCRs is activated by 5-HT, they exhibit distinct pharmacological properties (sensitivity to 5-HT, differential responsiveness to tryptamine, ergotamine and LSD).

### Serotoninergic GPCR expression is localized in particular cellular territories of *E*. *granulosus* protoscoleces

**Fig 6:**
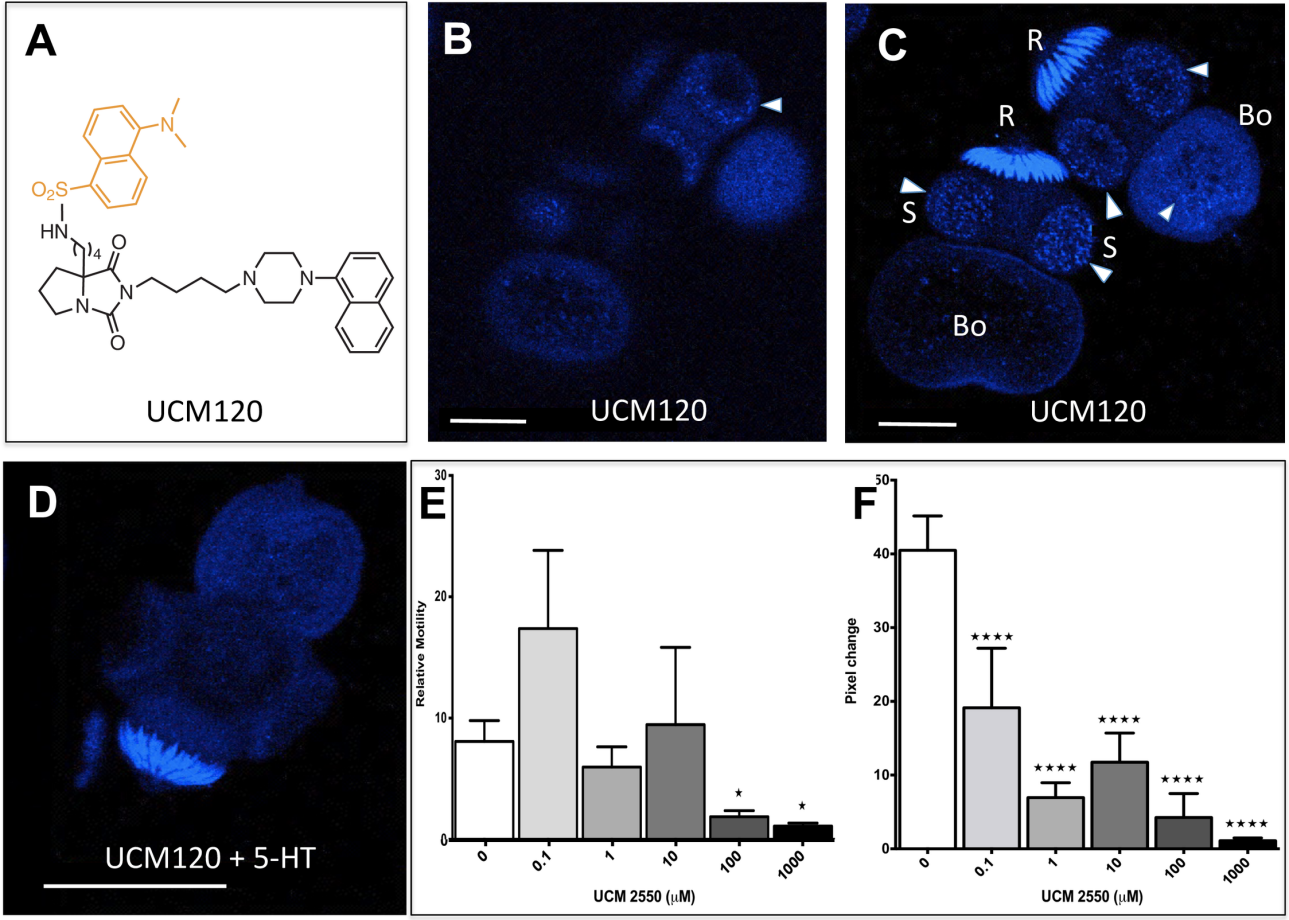
Protoscoleces of *Echinococcus granulosus* stained with the fluorescent probe UCM120 under the confocal scanning laser microscope and effect of the parent compound UCM2550 on the motility. **-** Protoscoleces were fixed, after washing they were incubated in the presence of the probe (100 μM) during several days, washed, fixed, washed again, mounted, and then observed by confocal microscopy. (A) Structure of the fluorescent probe UCM120 with the structure of the parent compound UCM2550 shown in black and the dansyl group shown in light brown. Images of two protoscoleces obtained from superficial (B) or from an entire stack of laser scanning (C). To assess specificity, protoscoleces were labeled under the same conditions with the probe (100 μM) in the presence of an excess (1000 μM) of 5-HT and the image projection was obtained from an entire stack of pictures (D). Relative motility observed in the presence of increasing concentrations of the parent compound UCM2550 in the WMicrotracker device measured after two hours of incubation (E); Image based motility quantification measured as a pixel change (F). Further details are provided in the Methods section. Asterisks indicate treatments found to be significantly different from the controls (*P ≤ 0.05) with t-test (E) or ANOVA and Dunnet post comparison tests (****P ≤ 0.0001, F). R: rostellum; S: suckers and Bo: body. The scale bar represents 50 μm in (A), (B) and (C) and 100 μm in (D). Arrowheads indicate localization of the probe.

In order to assess the localization of serotoninergic G-protein coupled receptors in larval stages of cestodes, we used a fluorescent probe with high affinity for mammalian serotoninergic GPCRs. Since the first attempts with *M*. *corti* tetrathyridia were unsuccessful and owing to the very limited availability of the probe, we concentrated our efforts in protoscoleces of *E*. *granulosus*, taking into account the higher size of *M*. *corti* tetratyridia compared to *E*. *granulosus* protoscoleces. The fluorescent probe—UCM120, a high affinity agonist of the human 5-HT_1A_ receptor (K_i_ = 2 nM, Fig 6A) [38]—displayed a distinctive staining pattern in protoscoleces of *E*. *granulosus*. A discontinuous and punctiform pattern of staining could be observed particularly concentrated in suckers but some of this pattern could also be observed in the body (Fig 6B and 6C). Several controls were performed in order to determine the specificity of pattern observed. When protoscoleces where incubated in the presence of the probe and an excess of serotonin (1 mM) was added, the staining pattern previously mentioned disappeared (Fig 6D). The hooks remained strongly stained as seen in controls (Fig 6D), suggesting this represented non-specific staining or autofluorescence. When protoscoleces where incubated in the presence of the non fluorescent analog UCM2550, motility was inhibited (Fig 6E, S2 Video). The dose-response curve obtained with the WMicrotracker device showed a significant fall of motility (relative to basal) starting from 100 μM (*p< 0.05) until the highest concentration tested of 1000 μM during a period of two hours (*p< 0.05; Fig 6E). However, using a video based method, lower concentrations produced significant inhibition of motility, starting from 0.1 μM (**** P ≤ 0.0001; Fig 6F).

## Discussion

This work provides for the first time, functional evidence for the existence of serotoninergic GPCRs in cestodes. Bioinformatic predictions suggesting the presence of several 5-HT-GPCR coding genes were validated by functional studies. From diverse genomic and transcriptomic databases, several gene models with putative seven transmembrane segments and amino acid identity with serotoninergic GPCRs were identified in *E*. *granulosus* and *M*. *corti*. The information obtained was used for the cloning of three putative 5-HT GPCRs of cestodes: 5-HT_7Egran1_, 5-HT_7Egran2_ and 5-HT_7Mco1_. The multiple sequence alignment of these clones with serotoninergic and other aminergic GPCRs suggests that the sequences obtained correspond to serotoninergic GPCRs. This result is principally supported by the multiple sequence alignment analysis of residues potentially involved in ligand binding in transmembrane domain 5. Many aminergic ligands form critical interactions with residues in TM5, in particular the residues 5.42, 5.43 and 5.46 [60, 62]. These residues can be used to discriminate between serotoninergic and other aminergic GPCRs and much of the specificity of GPCRs could be attributable to TM5 properties [68]. The crucial residues 5.42 and 5.46 are generally conserved in mammalian serotonin receptors, particularly in subtypes 1 (5-HT_1_) and 7 (5-HT_7_). It was reported that those residues are usually less polar in serotoninergic (no more than one polar residue) than the corresponding residues of adrenergic, dopaminergic and histaminergic receptors [62]. The substitution of the polar serine by the non polar alanine at position 5.42 observed here in cestode 5HT-GPCRs was reported before in some invertebrate receptors, for example in *D*. *japonica* [15] and *S*. *mansoni* [18]. Indeed, the residues “AA” in the position 5.42 and 5.46 respectively in planarian receptors were proposed as diagnostic for assignment to the clade 7 of the invertebrate receptors [15]. The threonine residue in position 5.43 is also conserved in cestode and trematode sequences as it was in mammalian receptors. In addition, several motifs commonly found in aminergic receptors, that could be critical for receptor function, were also found in the cestode sequences.

Phylogenetic analysis showed a close relationship between the cloned cestode receptors and the clade 7 according to the invertebrate classification. These subtype of receptors are coupled to Gs predicting an increase of cAMP levels upon stimulation. Consistent with the bioinformatic prediction, all the cloned cestode receptors produced high levels of cAMP upon incubation with 5-HT.

The cestode 5-HT GPCRs were found to be expressed in motile life cycle stages such as protoscolex and adult worms, as well as in the hydatid cyst, suggesting that those receptors could have other activities besides motor control.

The molecular modeling of the cloned receptors and the comparison with the mammalian counterpart showed a striking resemblance at the structural level, in coincidence with the results obtained by multiple sequence alignment. The position of the orthosteric binding pocket and the docking with ergotamine are in line with these structural similarities.

In agreement with bioinformatic predictions, the increase in larval motility upon incubation with serotonin suggests the presence of serotoninergic receptors. In concordance with previous work [3], the addition of serotonin to *M*. *corti* tetrathyridia caused a significant stimulation of motility in a dose response fashion. Previously, we have shown that *E*. *granulosus* protoscoleces responded to serotonin [16] using the same method. The stimulation of *M*. *corti* tetrathyridia motility exerted by serotonin was observed with higher concentrations (starting from 500 μM during 30 minutes of incubation, using a light scattering method) than those previously reported (100 nM, during 30 minutes of incubation) [3] and this could be attributed to differences in the biological samples used and/or the different sensitivity of the equipment employed in both cases. Other factors such as preincubation time and medium could also contribute to the differences observed. We were unable, by using imaging techniques, to find a significant stimulation of motility below 1000 μM of serotonin after 30 minutes of incubation with the drug. The increase on motility obtained in the presence of this neurotransmitter was observed until 60 minutes, but later the activity tended to fall. This could be due to an inactivation process after overstimulation of serotoninergic receptors [61].

In order to confirm the bioinformatic and the phylogenetic predictions, we heterologously expressed the cloned sequences and analyzed the intracellular response to receptor ligands. Remarkably, the three cloned sequences yielded elevated levels of cAMP accumulation upon incubation with serotonin. This response was not observed with other neurotransmitters. Interestingly, for two of the cloned receptors the EC_50_ was in the picomolar range. To the best of our knowledge, this is the lowest EC_50_ value reported for 5-HT GPCRs and would identify them as the most sensitive 5-HT receptors reported in any invertebrate. Differences between the three cloned receptors were also observed in their responses to three tested ligands: tryptamine was an activator of 5-HT_7Egran2_ and 5-HT_7Mco1_ but not 5-HT_7Egran1_; ergotamine activated the three GPCRs with variable efficacy, behaving as a low efficacy partial agonist at 5-HT_7Egran1_ and a higher efficacy partial agonist at 5-HT_7Egran2_ and 5-HT_7Mco1_; the hallucinogen LSD was a potent agonist [69] of 5-HT_7Egran1_ and 5-HT_7Mco1_ but an antagonist at 5-HT_7Egran2_. This differential response to LSD could be related to the divergence of 5-HT_7Egran2_ from 5-HT_7Egran1_ and 5-HT_7Mco1_ as seen in the multiple sequence alignment and phylogeny (Fig 1).

The EC_50_ for 5-HT in 5-HT receptors from invertebrates [18, 70, 71], as well as from the vertebrate host [72], show values in the nM to μM interval. Unexpectedly, the receptors cloned showed distinct pharmacological properties characterized by an extremely high sensitivity (picomolar EC_50_ value) to 5-HT in 5-HT_7Egran2_ and 5-HT_7Mco1_. Another remarkable difference with other receptors was that the allucinogen LSD, which has an agonist activity in several 5-HT7 receptors from invertebrates [73–76] and vertebrates [69], behaved as an antagonist in the 5-HT_7Egran2_ receptor. The unique properties of these highly expressed tapeworm receptors may be exploitable for drug design.

Another noteworthy discovery was a high level of basal coupling observed for two of the GPCRs, which could relate to their high affinity for serotonin (picomolar EC_50_ values). Basal coupling may have resulted from the presence of low levels of contaminating 5-HT in media in the face of the high receptor affinity for 5-HT. Alternatively, basal activity may represent constitutive activity of these receptors. There are several examples of constitutive activity in GPCRs of diverse ligand specificity [77], including serotoninergic GPCRs from vertebrates [78] and invertebrates [79]. The physiological significance of this basal coupling activity–if manifest outside the heterologous assays system used here—is not clear but constitutive activity of these GPCRs may provide tonic support for basal neuromuscular activity [77].

There is clear discordance between the concentration of serotonin needed to activate the receptors heterologously expressed *in vitro* (picomolar-nanomolar range) and the concentration to induce motility *in vivo* (micromolar range) in larval stages of cestodes. There are several characteristics of each assay system which could explain this difference. For example, in the *in vitro* system a high density of receptors are directly exposed to the media, while *in vivo* endogenous levels of receptors will be localized within internal compartments of the parasite. Rapid uptake of serotonin by endogenous cells could also contribute to the low potency of 5-HT on intact worms. Differences in receptor environment may also play a role, including interacting proteins such as G proteins with different affinities for these receptors in both systems. Finally, in the whole parasite several receptors subtypes are probably activated at the same time: some receptors will stimulate the cAMP generation by Gs coupling (e.g. 5-HT_7Egran1_ and 5-HT7_Egran2_ in *Echinococcus granulosus*) while others may decrease cAMP through Gi coupling [for example, receptors encoded by gene models ECANG7_00799 (named as 5-HT_1Ecan1_ in Fig 1) and ECANG7_02049 (named as 5-HT_1Ecan2_ in Fig 1) are yet to be characterized].

The fluorescent images obtained showed a particular pattern of punctiform and discontinuous staining concentrated in the suckers of the protocolex, structures involved in parasite attachment to the host intestine. The addition of excess 5-HT caused the disappearance of this signal and moreover, the addition of the analogous UCM2550 to protoscoleces in motility experiments, resulted in inhibition of motility supporting the results obtained by imaging methods. In this case, serotonin could be exerting its effects through serotoninergic receptors located at the surface of the protoscolex (particularly at the suckers) and in this way the parasite could sense the serotonin from the host.

Neuroactive substances such as serotonin could play a leading role not only at neuromuscular control but also in development and reproduction in flatworms [5, 80] and other invertebrates [81]. The precise role played by serotonin at the neuromuscular junction in flatworms is still unknown. In other invertebrates, for example at the crustacean neuromuscular junction, it was shown that the application of 5-HT increases the total synaptic pool size of releasable synaptic vesicles in response to an action potential in the presynaptic terminal [82]. Moreover, it was shown in the same system that 5-HT accelerates the kinetics with which the transmitter is released and increase the total amount of transmitter released [83]. Perhaps, it could be hypothesized that a similar mechanism of action could be operating in a similar way at neuromuscular junctions in cestodes after 5-HT addition but more experiments are needed to test this hypothesis. In *E*. *granulosus* it was found that serotonin could have a major role in development [16]. However, the potential role played in development by the GPCRs described here is at present unknown. The innervation of reproductive structures by the serotoninergic nerve elements could be also of major importance for parasite propagation [5]. In *E*. *granulosus*, the genital atrium and associated reproductive ducts are richly innervated with serotoninergic nerve cells bodies and nerve fibres [84]. For all these reasons, targeting the serotoninergic structures of the parasite will convey potential benefits beyond the essential function of parasite attachment: development and reproduction could also be impaired. Considering the particular sensitivity to 5-HT and their different pharmacology, we view the serotoninergic GPCRs described here are promising drug targets for *Echinococcus spp*. A more detailed characterization of the response of the receptors cloned here to other known serotoninergic ligands will advance our structural understanding of these binding sites to allow identification of selective cestode 5HT receptor antagonists.

Cloning and successful heterologous expression of these GPCRs now permits the possibility of high throughput screening campaign for the first time. The development of more specific agents against the parasitic over the mammalian host could deliver novel drugs for treatment of hydatid disease.

In conclusion, we have identified for the first time several genes encoding for serotoninergic G-protein coupled receptors from cestodes based on: i) seven transmembrane domains and conserved motifs commonly found on this kind of receptors, ii) striking structural similarity found by homology modeling, iii) high levels of cAMP accumulation in functional studies after heterologous expression of cDNAs in HEK-293 cells showing overall agreement with in vitro responses of parasites to serotonin and 5-HT GPCR ligands. The cloned receptors showed distinct pharmacological properties with differential sensitivity to 5-HT and differential responsiveness to tested ligands. As these genes are expressed in clinically relevant parasite stages, these GPCRs merit evaluation as novel targets for chemotherapy to combat neglected diseases caused by cestodes.

## Supporting information

S1 Table Primers used for serotoninergic GPCR cloning and first strand cDNA synthesis. (DOCX)

S1 Text Nucleotide and amino acid sequences of cloned 5-HT_7Egran1_, 5-HT_7Egran2_ and 5-HT_7Mco1_ cestode 5-HT receptors. (DOCX)

S1 Fig Ramachandran plots of the three modeled G-protein coupled receptors from cestodes. General, glycine, pre-pro and proline plots of the models for: A) 5-HT7Egran1, B) 5-HT7Egran2 and C) 5-HT7Mco1. The difference between favored and allowed regions are indicated by the color intensity. Residues are indicated in blue (general, pre-pro), brown (glycine) and green (proline). Residues in outlier regions are indicated in white.
(TIF)

S2 Fig Diagram of the imaging technique used for protoscolex and tetrathyridium motility quantification. Major steps in the processing of video images are shown. Further details are provided in the material and methods section.
(TIF)

S3 Fig Multiple sequence alignment of serotoninergic G-protein coupled receptors with *Echinococcus granulosus* and *Mesocestoides corti* GPCRs. A Multalin alignment was performed using the three amino acid sequences encoding for 5-HT GPCRs of cestodes (5-HT_7Egran1_, 5-HT_7Egran2_ and 5-HT_7Mco1_) cloned in this work and representative examples of vertebrate and invertebrate serotoninergic GPCRs: Human 5-HT1B receptor (5HT1bHs, NP_000854.1) and 5-HT7 receptor (5-HT7Hs, P34969.2); *Caenorhabditis elegans* ser-4 (CeleSER_4, NP_497452.1) and ser-7 (CeleSER_7, NP_741730.1); *Schistosoma mansoni* 5-HT7Sm receptor (ANG84010.1). Amino acid residues which are identical in all the aligned sequences are shown in white on black background, residues identical in at least six of the aligned sequences are shown in white on dark gray background, and finally, residues identical in five of the aligned sequences are shown black on light gray background. Transmembrane domains (TM1–TM7) are shown as thick lines above the alignment and extracellular (EL) or intracellular (IL) loops are indicated. The most important residues involved in ligand recognition or receptor function were indicated inside boxes. The simbols C1 and C2 marked in bold inside boxes, refers to residues V155 and L6.37 respectively, potentially important for coupling with G-proteins. Conserved motifs were also indicated below the alignment.
(TIF)

S4 Fig Multiple sequence alignment of the fifth transmembrane segment of cloned cestode serotoninergic GPCRs with other members of the rhodopsin family of GPCRs. The multiple sequence alignment of the fifth transmembrane segment of 5-HT_7Egran1_ with other GPCRs is shown in A). In B), the same alignment is shown but the cestode sequence shown in A) was replaced with 5-HT_7Egran2_ and finally, in C) the cestode sequence of 5-HT_7Mco1_ was used instead. Transmembrane domain five (TM5) is shown as a thick line above the alignment. Amino acid residues which are identical in all the aligned sequences are printed in white on black background, residues identical in at least seven of the aligned sequences are printed in white on gray background, and finally, residues identical in at least five of the aligned sequences are printed black on gray background. Residues important for function were underlined and indicated by an arrow below the alignment. The sequences used for each alignment were (with accession numbers in parenthesis): Sm5HTR from *Schistosoma mansoni* (ANG84010.1) and the rest of the sequences were from *Homo sapiens*: 5HT1B_HUMA, 5-HT1B receptor (NP_000854.1); 5HT2A_HUMA, 5-HT2A receptor (P28223); 5HT7R_HUMA, 5-HT7 receptor (P34969); ADA1A_HUMA, Alpha-1A adrenergic receptor (P35348); D2_HUMAN, Dopamine receptor D2 (A0A024R3C5); ACM1_HUMAN, Muscarinic acetylcholine receptor M1 (P11229) and HRH1_HUMAN, Histamine H1 receptor (P35367).
(TIF)

S5 Fig Gene expression levels of two serotoninergic GPCRs. Levels of expression (in RPKM) of 5-HT_7Egran1_ and 5-HT_7Egran2_ serotoninergic GPCRs in the oncosphere, cyst, protoscolex and adult stage of *Echinococcus granulosus*.
(TIF)

S6 Fig Serotonin (5-HT) stimulates the neuromuscular activity of the tetrathyridium of *Mesocestoides corti*. A) Channel activity traces from the WMicrotracker device before (Basal) and after the addition of 2000 μM of serotonin (5-HT 2000 μM) during a period of two hours. B) Relative motility counts without (0 μM, white bar) or after the addition of increasing concentrations of serotonin during a period of two hours. C) Relative motility counts after the addition of the same concentrations of serotonin as in B), but accumulated during a period of 30 minutes and during the intervals of 0–30 (30), 30–60 (60), 60–90 (90) and 90–120 (120) minutes after the addition of 5-HT. D) Relative motility without (0 μM, white bar) or after the addition of increasing concentrations of serotonin during a period of 30 minutes by video imaging. ANOVA and Dunnet post comparison tests were performed. Asterisks indicate treatments found to be significantly different from the controls (*P ≤ 0.05, **P ≤ 0.01 and ***P ≤ 0.001). Most of the experiments were performed using 16 technical replicates per treatment condition and repeated with four different biological samples with the exception of the experiment showed in D, in which eight technical replicates and repeated with three different biological samples.
(TIF)

S1 Video Video images of the *Mesocestoides corti* tetrathyridium in RPMI medium alone (control) or with 1000 μM of serotonin in RPMI. Further details are provided in the Methods section.
(MOV)

S2 Video Video images of *Echinococcus granulosus* protoscoleces in RPMI medium alone (control) or with 100 μM of UCM2550 in RPMI. Further details are provided in the Methods section.
(MOV)
