# Supplementary material for: Unique pharmacological properties of serotoninergic G-protein coupled receptors from cestodes
Source: PLoS Negl Trop Dis. 2018 Feb 9;12(2):e0006267. doi: 10.1371/journal.pntd.0006267 (PMC5823469; doi:10.1371/journal.pntd.0006267)
Supplement: S1 Table — (DOCX) [file pntd.0006267.s001.docx]

Table 1: Primers used for GPCR cloning

| Primer name | Sequence (5´to 3´) | Amplicon size (in bp) | Product obtained |
| --- | --- | --- | --- |
| 6088 Frw | TATTTTCAGGGAGAATTCCCGGGTATGGATCTACTTATCAACCG | 2100 | 5-HT_7Ecan1a_ |
| 6088 Rv* | GCGAGGCAGATCGTCAGTCAGTCACTATGGATTTGCTACACTAAG |  |  |
| 6092 Frw | TATTTTCAGGGAGAATTCCCGGGTATGGATACAAATGTATCTACTTC | 1731 | 5-HT_7Ecan2_ |
| 6092 Rv* | GCGAGGCAGATCGTCAGTCAGTCACTATTCGGTTAGCATGGAACTG |  |  |
| 4401 Frw | TATTTTCAGGGAGAATTCCCGGGTATGGCTACTCAACTCAATG | 2091 | 5-HT_7Mco1_ |
| 4401 Rv* | GCGAGGCAGATCGTCAGTCAGTCATCATTCCCATTCAATTAACC |  |  |

*Also used for first strand cDNA synthesis.
