# Supplementary material for: Unique pharmacological properties of serotoninergic G-protein coupled receptors from cestodes
Source: PLoS Negl Trop Dis. 2018 Feb 9;12(2):e0006267. doi: 10.1371/journal.pntd.0006267 (PMC5823469; doi:10.1371/journal.pntd.0006267)
Supplement: S1 Text — (DOCX) [file pntd.0006267.s002.docx]

>5-ht7egran1

ATGGATCTACTTATCAACCGATCCATCCCAGAGGAGGCATTTCTTGCTGCATGCAAACCTCATGCAGAGATCGACGTTAAAAGCATCATTCTTGCAATTGTCCTCGGTCTCCTCCTCCTCGGCACGGCGGGCGGAAATATGCTAGTCATTATAGCTATTCTGATTGTGAAAAAATTACGCTCTCCCACAAACCTACTCATTGTAAATCTCGCAGTGACCGACTTTCTTGTCAGTATTCTTGTCCTTCCATTTGCTATTGCATATCAAATCCTCGGTTATTGGCCTTTCAATCAAATCATCTGCGATCTTTACAGTCTCTCCGATGTGTTGCTTTGCACACTGTCGATTCTTAGTCTCTGTACAATCTCAATTGACCGATATTTGGCCATTACGAAGCCCCTTCAGTACGCCGCCAAACGAACACCAAAACGAATGTTGATAATGATCATCATCTCCTGGCTCCTCTCTGCTGCAATTAGTATTCCACCTGTATTCGGTTGGGAGCAGAAAAACAGCCCCTTCTACTGTGGTTACAGTGAGGAGCTGACCTACCAGATCTATGCAACCATGACAGCTTTCTACATTCCTTTGACAGTGATGCTAGTCCTCTATGGGAAAATATTAGTTCTGGCTAAGCAAATGGCCTCAGTGGATGCACAGGTAGGACGCAAGGGTAGTGTCGACACACAGACCCGATCGTCTTCCATTCCAGAGTGGAATAGAAGTTCCCTTTATGCAGCTGGAGAGAGCTACAAGGCTTCAGCACTTTATCCCGATTTAAAGAAGGAGCATAAGGGAAATAGAGGCGATTCGAAGCCGCGGCAAAGTGTGGTTATTTTTAAGCCTACCCCAGTGGTGATGCGAATGGATAGGCGACCATATAGAGGAAGGAAGATTAACAGTTCGCCATCAGCGCTTGGGAATAATGGTAGATCCTCGGAGGTGAGCGGGAGTAGTAGAAGTTTGTACGCATATGCCAAGTCCCCCTTGTCCATGCTTCGATACCACAGAAGACGCAAACCCTCGGATCAGGGAAAGGGGGAAACGCACAAGGCGGTCACCACACTTGGAGTTATTATGGGATGCTTTACCATTTGCTGGCTGCCATTTTTTGTTTCTCAGCTTATAACTCCAATAATCAACTGTTTCACAGAGCAAAGATTCTCCATTCCCCCTACCCTCTTTCAGGTGTTCATCTGGCTAGGCTATGGAAACAGTTTCCTAAATCCTCTCATTTACGCGCTATTTAACCGAGAATTTCGATTGCCATTTCTCTACATTCTTCGACTACAATGCAATGATATAAACACTCGACTCCGAACTGAAACCTTTTCTCATCAATTCGGGCTGCCACAAAAATCCAGAGTGGGTTATAGCAACTCCCTTTCCCGTGGGAGCTCCAGTGTCTCAGGAAGTAAACGTCAACGACGACAGAAACCGCGTGGACTGAGTCCTTTGACCAATTCCCAAGTCCAAACATCTCCGAGTAAAACTAGCCAGGTAGCTGAAGCTTCAAATGTAGAGATTGAAAAGCCCCCACTCCGCTCAAGTTTAAACCTCAATACAGTGGCTCCTGAGGAGCCCAAACCTCTTCCGGTTCAACCTCCCAACAATCTGCCCTCCCCCCTTCATCTCACTCCACTTCAACCTAACACACCATTGCAGAAGGGCGATCTGCTAGAGAACAATCACCCACCACCCTCGTGGCTGATGAGTCGACGAGAGAGCCTTGCCAGTATCCGAAGCAATTACGACACCTACATACCAAATCTAAATGCGGAATTCCAAAGAAGTCCACTCCCCACCCCATCCAGTGCTCCAGCCTACTTGACTCCGTCTATGAACAATGTGAAAATGTGGACGAAGGCACGGGAGTTTCGACCTAATCAAGTGCCACCCGAGAGGTTCCACAGATGGTGTGAAAACATATCGTGTACAGGTGTTAAAAGCAACTCCTCCCTTAGTGTAGCAAATCCATAG

>5-ht7egran2

ATGGATACAAATGTATCTACTTCGGTGCTTGCAGCTACCCAAGTCCCCAGCACTGCCGCCCTAGGTTCAATCCCTCTTGCAGTTACTTTCTGTATCATCACTGCTCTAACTATTCTAGGCAACACATTAGTTATCTCTGCTGTCCTTCTAGTACACAAACTTCGGTGTCCTAGCAATTTTCTGATCGTCAGTCTGGCTGCTAGTGATCTGATGGTCAGCATTATAGTGATGCCTTTCAGCACCTACTTGGAATATCGGCGATACTGGGACCTAGGTGAAGTTGCTTGTGACATATTTATCGTTTTCGATGTTTTCCTATGCACAGCCTCCATTTTGAATCTTTGTGCTATCAGTATTGACCGATATCTTGCAGTTACCCGACCATTTGAGTACGTGTACAAACGGACTCCTAAGCGAATGCTGATAATGATTCTCACGGCTTGGTCAATTTCAGCATTAATAAGCATCCCCCCGACATTTGGTTTCAAAGATGAATTTGTTCCTGGGAAATGCGCCTATAGTCAAAATTTTATTTATCAAATCTATGCTTGCTTCGGTGCTTTTTACATACCTCTTATCGTCATGCTAATACTATACGGCCGGATCGTAATTTTAGCTAGAAGGATTGTAAAATCTGACCGAATCAGATTACCATCAAAACCTGGTGATGAAAAACGAAATTCAATTCAAAATTACCCTGAATCACAGGAAACTGATGAAGGCGCCTCCGAGGATAAAAGCAAATTTACCTATCCATGCTTTGGCAAAACCTGCTGTCTTTACTTACGCAAACGGGAATCTTTTGATTCTACCGACAAGTCTGTGGATCCTAATTTCTTTAGCAATGTCTCTTCTATTCATTCCGTTCCCACCGCACCAGTTGTATGTATTAGTGAAGCAGGTGATAGTCCCCTACTCAATCCCCCCCTCATTAAAGAAAACAACTGCCCACCTGACCCAGTTCATCACTTCAGCCGTCTGCCTCCCCAAAAATCCTGCCCTAATAGCAAACTGAGAGAGCACCCAAATAACGGCTGTGAGCGTTCATCTCCGGGAGTTGAGTTCCTTGAACCATCTACTCAAAGACCGCAGAGCCTCTTCACGAACCAGCATCGAGTAAGCTTGCATCCTGGCGCAGGAAATCTCAAGCATCGCTCCTCACTGTTACGCGATCGAGCCTACTCTGTCACTGTTGCGCACATACAAACAAAAAACCCGCGAAGCAGCTTATTCTTCTTCTCCAAACAGCGTCTCAGCATTGCTCTCCACTTAAAACGACCAGGAATTAAACGCTCAAATGAGGCCAAAGCCATCCGAACCCTTGGCGTCATTATGGGTGTTTTCTGCATTTGCTGGCTACCATTTTTCATCGTTGCGCTGGGTCGACCTCTCTACAATTACATACATAACACAGAAAAGGATATTGACCCCCGTCTCAATTGCTTCTTCCTCTGGCTAGGCTACGTGAACTCGGCACTTAACCCCTTAATCTACGCTATCTTCAACCGTGAATTTCGTCGACCCTTCTGGGAGTTGATGTCATGCCATTGCTTGAACATTAACGCGCGTCTTCGTGAACGTCGCTACCAGCATGAGTACAGACCTCCACCCGTCCCCTTACCAAACATTTCTGGTGGCGGTGGCACTGAAGGTGAAACGCCCTCTCTTTCCCGATCTCGAAATTCTGCTCATTTGCTTGACAGAAGACACAGTTCCATGCTAACCGAATAG

>5-ht7mco1

ATGGCTACTCAACTCAATGACCCCTCCATCACATTTAAGGCCTGGGGATGGAGTGAAAACTGCACCCTTCTTCTTGATCAACTCGTCAATAGGACAATCTCTGAGGCTTTTTTCCTTGAGGCGTGTAAACCTCATGCAGACATCGACGCTGGAACAGTTGTCCTCGCAATCCTCCTTACACTTCTCCTTCTAGGAACGGCGGGAGGAAATATTTTGGTCATTATAGCTATCTTGATTGTGAAAAAACTCCGGTCACCCACAAACCTGCTCATTGTCAACCTCGCAGTGACGGACTTTCTTGTCAGCATACTAGTCTTGCCGTTCGCAATCGCCTACCAAATTTTAGGCTACTGGCCATTTAATCAGATCATCTGTAATCTCTACAATATCTCAGATGTCCTTCTATGCACCCTATCGATTCTGAGCCTTTGCACAATTTCCATTGACCGTTATCTTGCCATTACGAAACCTCTTCAATACGCTGCTAAGAGGACGCCTAAACGGATGCTGCTAATGATTTGTATCTCGTGGTTACTATCCGCCGCAATTAGCATTCCACCCGTATTTGGATGGGAGCAAAAAAATAGTCCATTCTATTGCGGCTACAGTGAAGAATTAACCTACCAAATCTATGCAACAATGACGGCTTTTTATATTCCCCTTACAGTCATGGTTATCCTCTATGGAAAGATTTTGGTACTTGCCAAGCAAATGGCCCTTACGAATGCACAGGTCGCTCGCGAAAGCAGCACTGAGATGCAGGCAAGAACATCATCGATTCCTGCGTACGCTGACTGGAACAGGAATTCTCTCTATCCCACTAGAGAAAGCTACACATCTCAAAAGGTGTACACTGAGGCAAATGATAACTGGCAACAGATACTGGAGCCTAATAATACTGATTTCAAATCCCGACAAAGCGTTGTCAGTTTTAAGTCCCCTCCTGCGATAAGGCCACCGGGAAGATGCCAAGATGTGCGAGGAAAATTTTGCTGTCTGTTTGCGTCGTCTCATAGAGCCTCGACGGAAATTACCAATAACGGCAAGTCTTTAGAACAGACGGCCAGCCTTAGCGGTCCTACCAGACCCCCATTATCTTTGCTCCGCAGAAAGAGGAGACGCTCGCCCTCCGACCAAGGCAAGGGGGAAACGCATAAAGCAGTCACCACACTCGGAGTTATCATGGGATGCTTTACCGTCTGCTGGTTACCGTTTTTTGTGTCACAGCTCATCACTCCTATCATCAATTGCTTTACAAAGCAAAAGTTCTCTATTCCTCCGACCCTCTTTCAGGTGTTTGTTTGGCTCGGGTATTGCAACAGCTTTCTAAATCCGTTGATATACGCACTGTTCAACCGAGAGTTTCGTTTGCCCTTCATCTACATTCTTCGATTCCAATGCTACAGCATAAATACTCGGCTTCGAACTGAAACATTTTCACATCAGTTCGATCTGCCCAGAAAATCAGGACTAAGCTACCGTAACTCCCTCGCTCGTGGAAGCTCCAGCAGTTGTAATCGACGCCAGCAGCGACGCAAACTACGAGGTCTCAGTCCCTTGGCAAATGTCTCTCCACACGCAACAATAAACGCAGCTGGCCACACCAAGGCTCCTAGTTCGGAACACAACGTGTTCGTTTCACATCTGAGCTCGGAGCATGATGAAGTACTCCAAACCCCACACTCTCTCTTCGACCAGCCTAGCCGGTTCTCTATCCCAACGTCATTGTTCACTGCTTCGGAAGTTTTGGCCAATCCCAGTGGCAACGACCTCCTTGAAAACACACTCCGAATATGCCCTCTGCCTAACTGCAGTCAGGAGAGTGACGTGGGAATTCGAAGTGATAGGATATACGACACGCACATACCCTATCTGAATGTGCAGTTTCAGCGACGTCCGCGTAGCAGCCCCTCAAGTGCACCACCGAATTTGACTACCTTCGAAAACAGCCTGCACACGTGGACCGGAGTTAGTGACTACCAACCGAAGTCGCAAACGTGCGAGCGACGTCGAAGACAGTCAGATGCTGCAGTCTCCCCTCTTATGAGAAATAAGTTCCAAAAAAAACCACGGTTAATTGAATGGGAATGA

>5-HT_7Egran1_

MDLLINRSIPEEAFLAACKPHAEIDVKSIILAIVLGLLLLGTAGGNMLVIIAILIVKKLR SPTNLLIVNLAVTDFLVSILVLPFAIAYQILGYWPFNQIICDLYSLSDVLLCTLSILSLC TISIDRYLAITKPLQYAAKRTPKRMLIMIIISWLLSAAISIPPVFGWEQKNSPFYCGYSE ELTYQIYATMTAFYIPLTVMLVLYGKILVLAKQMASVDAQVGRKGSVDTQTRSSSIPEWN RSSLYAAGESYKASALYPDLKKEHKGNRGDSKPRQSVVIFKPTPVVMRMDRRPYRGRKINSSPSALGNNGRSSEVSGSSRSLYAYAKSPLSMLRYHRRRKPSDQGKGETHKAVTTLGVIMGCFTICWLPFFVSQLITPIINCFTEQRFSIPPTLFQVFIWLGYGNSFLNPLIYALFNREF RLPFLYILRLQCNDINTRLRTETFSHQFGLPQKSRVGYSNSLSRGSSSVSGSKRQRRQKP RGLSPLTNSQVQTSPSKTSQVAEASNVEIEKPPLRSSLNLNTVAPEEPKPLPVQPPNNLP SPLHLTPLQPNTPLQKGDLLENNHPPPSWLMSRRESLASIRSNYDTYIPNLNAEFQRSPL PTPSSAPAYLTPSMNNVKMWTKAREFRPNQVPPERFHRWCENISCTGVKSNSSLSVANP

>5-HT_7Egran2_

MDTNVSTSVLAATQVPSTAALGSIPLAVTFCIITALTILGNTLVISAVLLVHKLRCPSNF

LIVSLAASDLMVSIIVMPFSTYLEYRRYWDLGEVACDIFIVFDVFLCTASILNLCAISID

RYLAVTRPFEYVYKRTPKRMLIMILTAWSISALISIPPTFGFKDEFVPGKCAYSQNFIYQ

IYACFGAFYIPLIVMLILYGRIVILARRIVKSDRIRLPSKPGDEKRNSIQNYPESQETDE

GASEDKSKFTYPCFGKTCCLYLRKRESFDSTDKSVDPNFFSNVSSIHSVPTAPVVCISEA

GDSPLLNPPLIKENNCPPDPVHHFSRLPPQKSCPNSKLREHPNNGCERSSPGVEFLEPST

QRPQSLFTNQHRVSLHPGAGNLKHRSSLLRDRAYSVTVAHIQTKNPRSSLFFFSKQRLSI

ALHLKRPGIKRSNEAKAIRTLGVIMGVFCICWLPFFIVALGRPLYNYIHNTEKDIDPRLN

CFFLWLGYVNSALNPLIYAIFNREFRRPFWELMSCHCLNINARLRERRYQHEYRPPPVPL

PNISGGGGTEGETPSLSRSRNSAHLLDRRHSSMLTE

>5-HT_7Mco1_

MATQLNDPSITFKAWGWSENCTLLLDQLVNRTISEAFFLEACKPHADIDAGTVVLAILLT

LLLLGTAGGNILVIIAILIVKKLRSPTNLLIVNLAVTDFLVSILVLPFAIAYQILGYWPFNQIICNLYNISDVLLCTLSILSLCTISIDRYLAITKPLQYAAKRTPKRMLLMICISWLLSAAISIPPVFGWEQKNSPFYCGYSEELTYQIYATMTAFYIPLTVMVILYGKILVLAKQMALTNAQVARESSTEMQARTSSIPAYADWNRNSLYPTRESYTSQKVYTEANDNWQQILEPNNTDFKSRQSVVSFKSPPAIRPPGRCQDVRGKFCCLFASSHRASTEITNNGKSLEQTASLSGPTRPPLSLLRRKRRRSPSDQGKGETHKAVTTLGVIMGCFTVCWLPFFVSQLITPIINCFTKQKFSIPPTLFQVFVWLGYCNSFLNPLIYALFNREFRLPFIYILRFQCYSINTRLRTETFSHQFDLPRKSGLSYRNSLARGSSSSCNRRQQRRKLRGLSPLANVSPHATINAAGHTKAPSSEHNVFVSHLSSEHDEVLQTPHSLFDQPSRFSIPTSLFTASEVLANPSGNDLLENTLRICPLPNCSQESDVGIRSDRIYDTHIPYLNVQFQRRPRSSPSSAPPNLTTFENSLHTWTGVSDYQPKSQTCERRRRQSDAAVSPLMRNKFQKKPRLIEWE
