# Supplementary figures and images for: Unique pharmacological properties of serotoninergic G-protein coupled receptors from cestodes
Source: PLoS Negl Trop Dis. 2018 Feb 9;12(2):e0006267. doi: 10.1371/journal.pntd.0006267 (PMC5823469; doi:10.1371/journal.pntd.0006267)

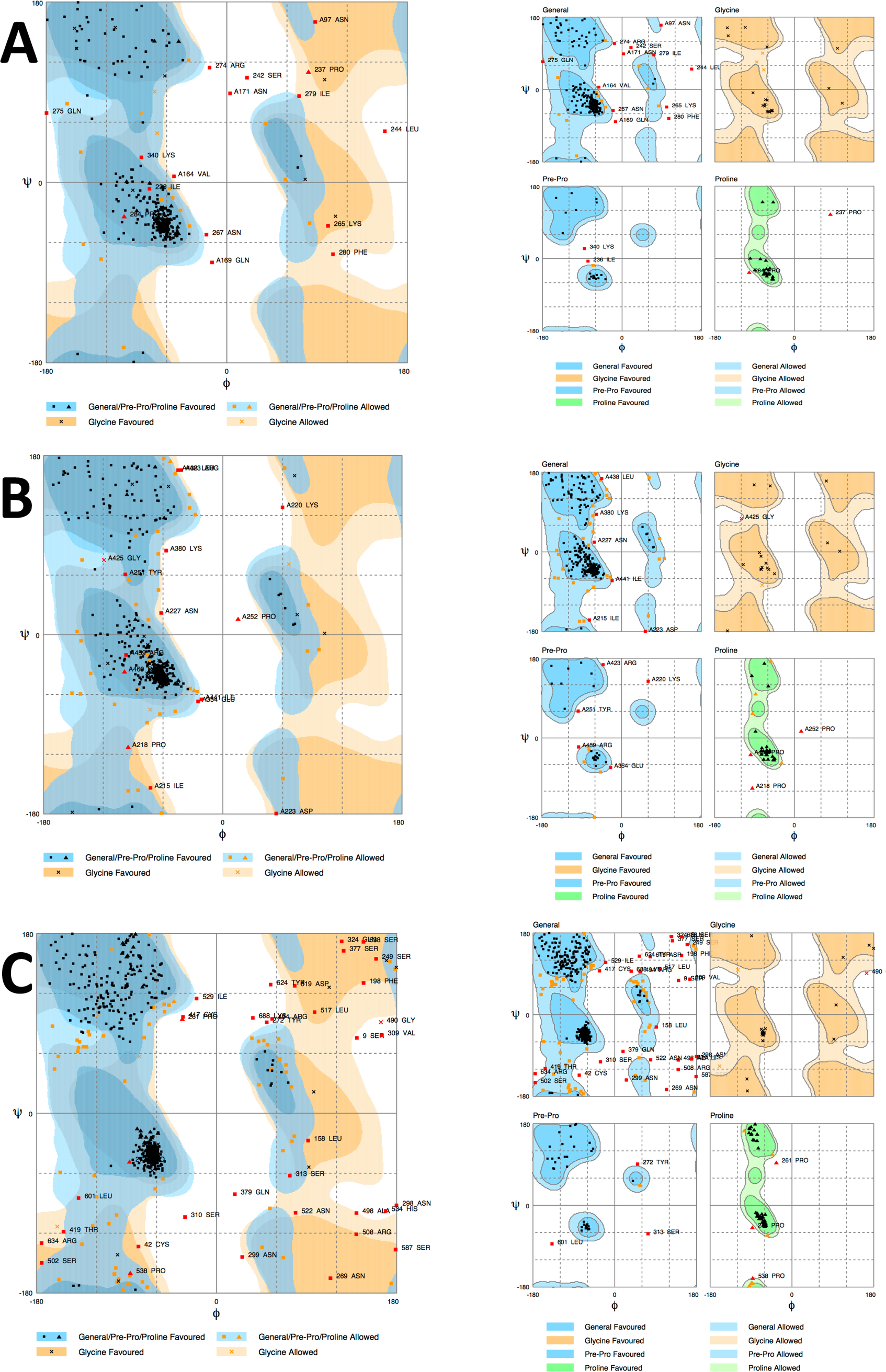

Supplement: S1 Fig — General, glycine, pre-pro and proline plots of the models for: A) 5-HT7Egran1, B) 5-HT7Egran2 and C) 5-HT7Mco1. The difference between favored and allowed regions are indicated by the color intensity. Residues are indicated in blue (general, pre-pro), brown (glycine) and green (proline). Residues in outlier regions are indicated in white. (TIF) [file pntd.0006267.s003.tif]

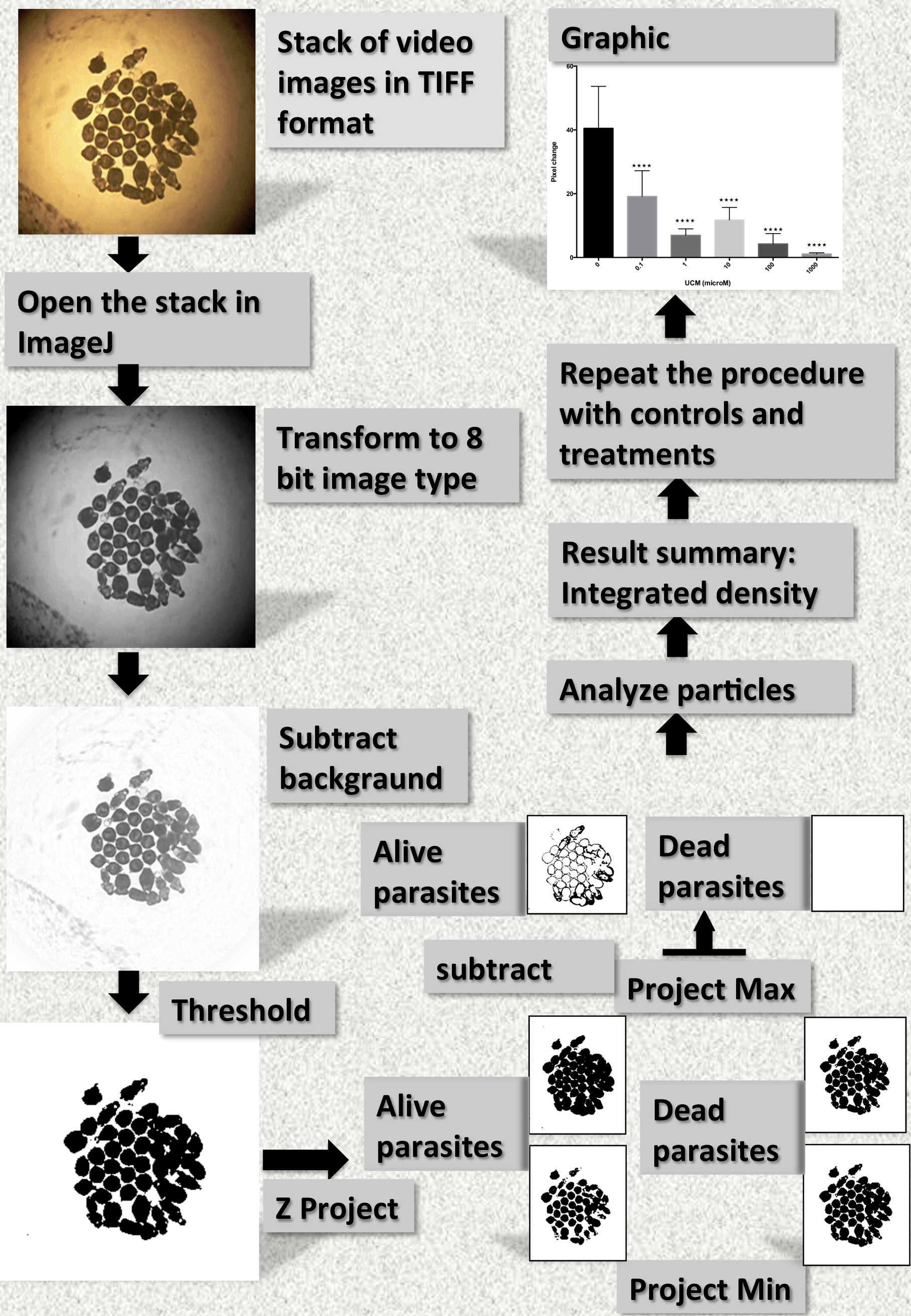

Supplement: S2 Fig — Major steps in the processing of video images are shown. Further details are provided in the material and methods section. (TIF) [file pntd.0006267.s004.tif]

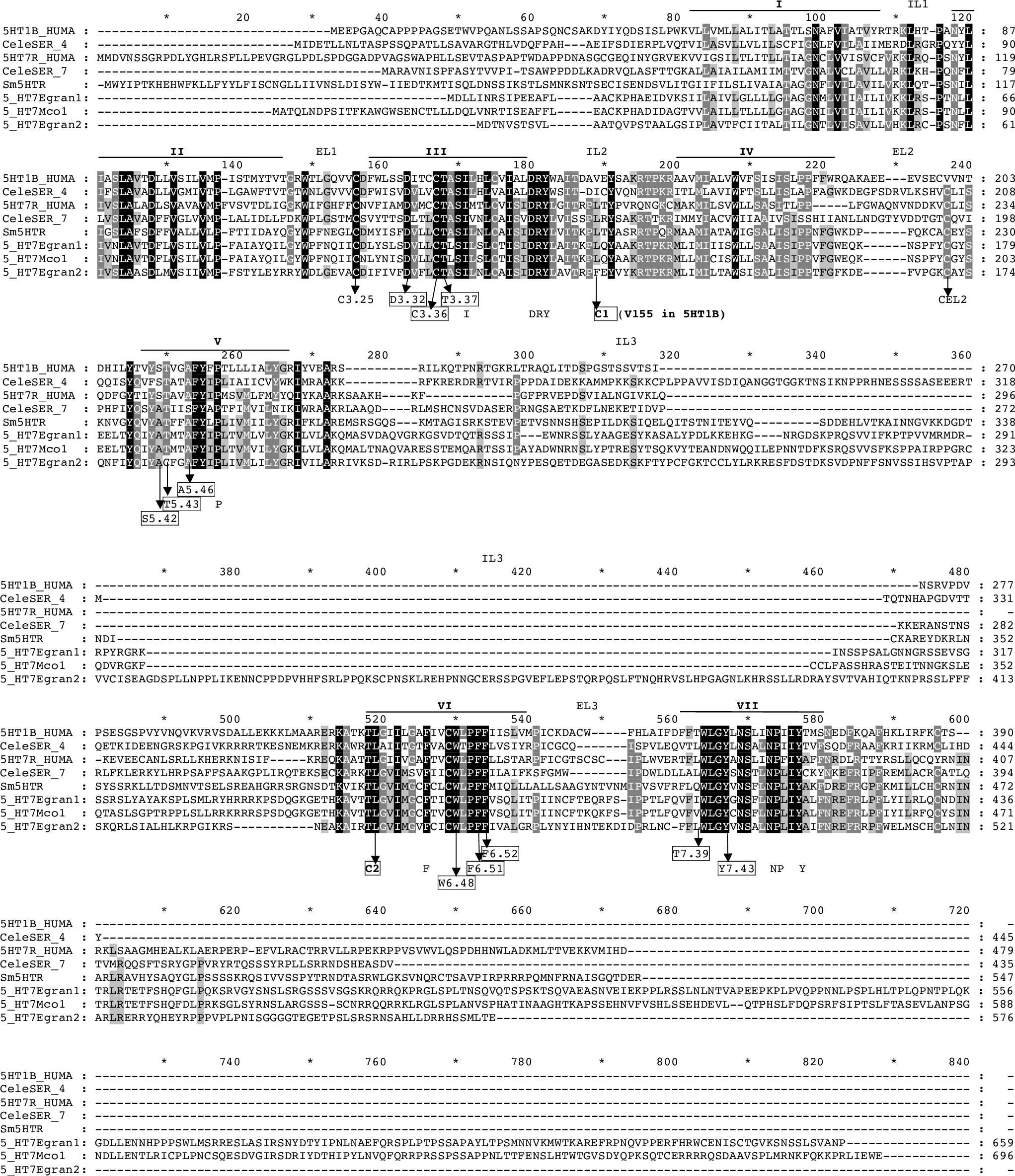

Supplement: S3 Fig — A Multalin alignment was performed using the three amino acid sequences encoding for 5-HT GPCRs of cestodes (5-HT7Egran1, 5-HT7Egran2 and 5-HT7Mco1) cloned in this work and representative examples of vertebrate and invertebrate serotoninergic GPCRs: Human 5-HT1B receptor (5HT1bHs, NP_000854.1) and 5-HT7 receptor (5-HT7Hs, P34969.2); Caenorhabditis elegans ser-4 (CeleSER_4, NP_497452.1) and ser-7 (CeleSER_7, NP_741730.1); Schistosoma mansoni 5-HT7Sm receptor (ANG84010.1). Amino acid residues which are identical in all the aligned sequences are shown in white on black background, residues identical in at least six of the aligned sequences are shown in white on dark gray background, and finally, residues identical in five of the aligned sequences are shown black on light gray background. Transmembrane domains (TM1–TM7) are shown as thick lines above the alignment and extracellular (EL) or intracellular (IL) loops are indicated. The most important residues involved in ligand recognition or receptor function were indicated inside boxes. The simbols C1 and C2 marked in bold inside boxes, refers to residues V155 and L6.37 respectively, potentially important for coupling with G-proteins. Conserved motifs were also indicated below the alignment. (TIF) [file pntd.0006267.s005.tif]

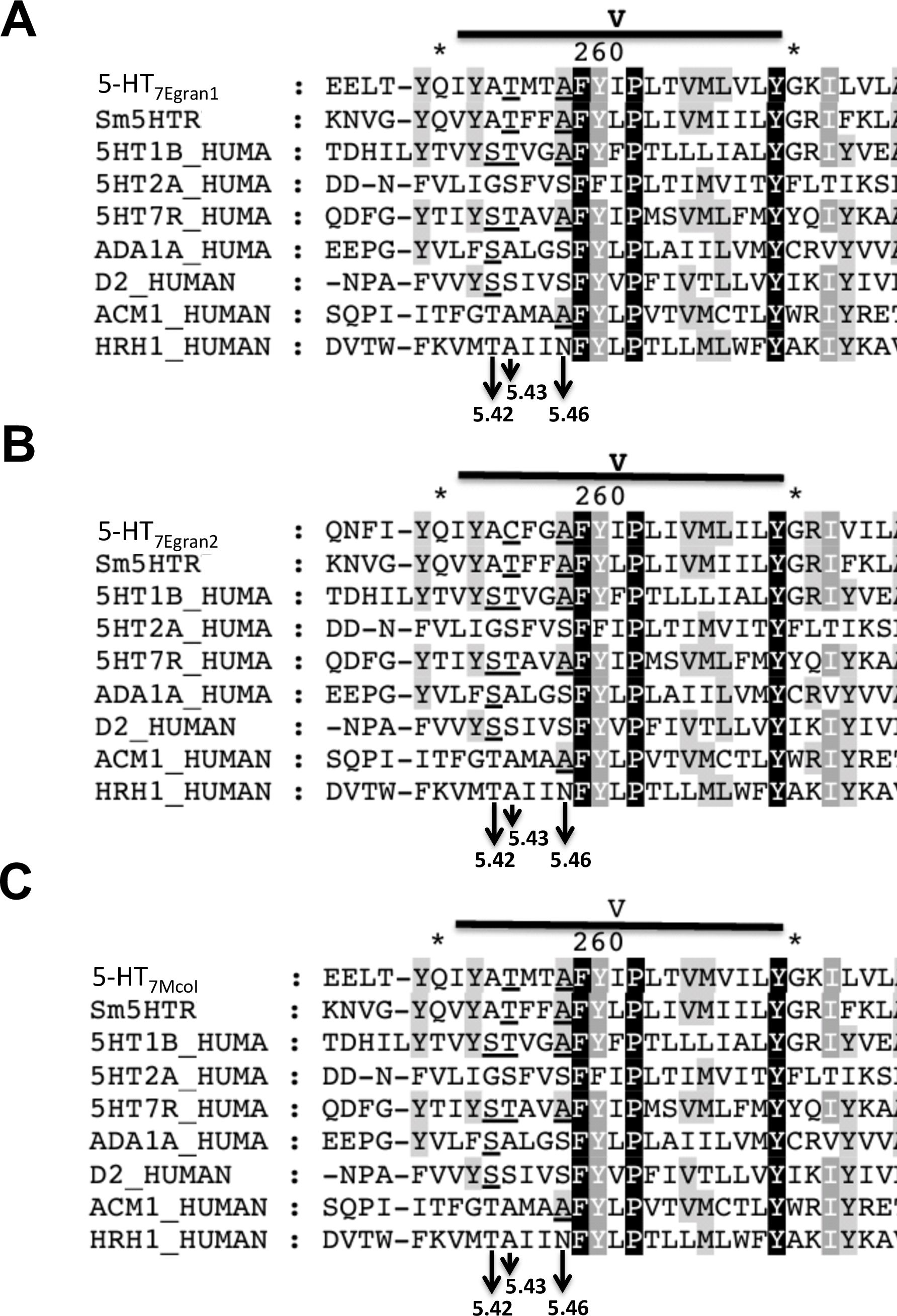

Supplement: S4 Fig — The multiple sequence alignment of the fifth transmembrane segment of 5-HT7Egran1 with other GPCRs is shown in A). In B), the same alignment is shown but the cestode sequence shown in A) was replaced with 5-HT7Egran2 and finally, in C) the cestode sequence of 5-HT7Mco1 was used instead. Transmembrane domain five (TM5) is shown as a thick line above the alignment. Amino acid residues which are identical in all the aligned sequences are printed in white on black background, residues identical in at least seven of the aligned sequences are printed in white on gray background, and finally, residues identical in at least five of the aligned sequences are printed black on gray background. Residues important for function were underlined and indicated by an arrow below the alignment. The sequences used for each alignment were (with accession numbers in parenthesis): Sm5HTR from Schistosoma mansoni (ANG84010.1) and the rest of the sequences were from Homo sapiens: 5HT1B_HUMA, 5-HT1B receptor (NP_000854.1); 5HT2A_HUMA, 5-HT2A receptor (P28223); 5HT7R_HUMA, 5-HT7 receptor (P34969); ADA1A_HUMA, Alpha-1A adrenergic receptor (P35348); D2_HUMAN, Dopamine receptor D2 (A0A024R3C5); ACM1_HUMAN, Muscarinic acetylcholine receptor M1 (P11229) and HRH1_HUMAN, Histamine H1 receptor (P35367). (TIF) [file pntd.0006267.s006.tif]

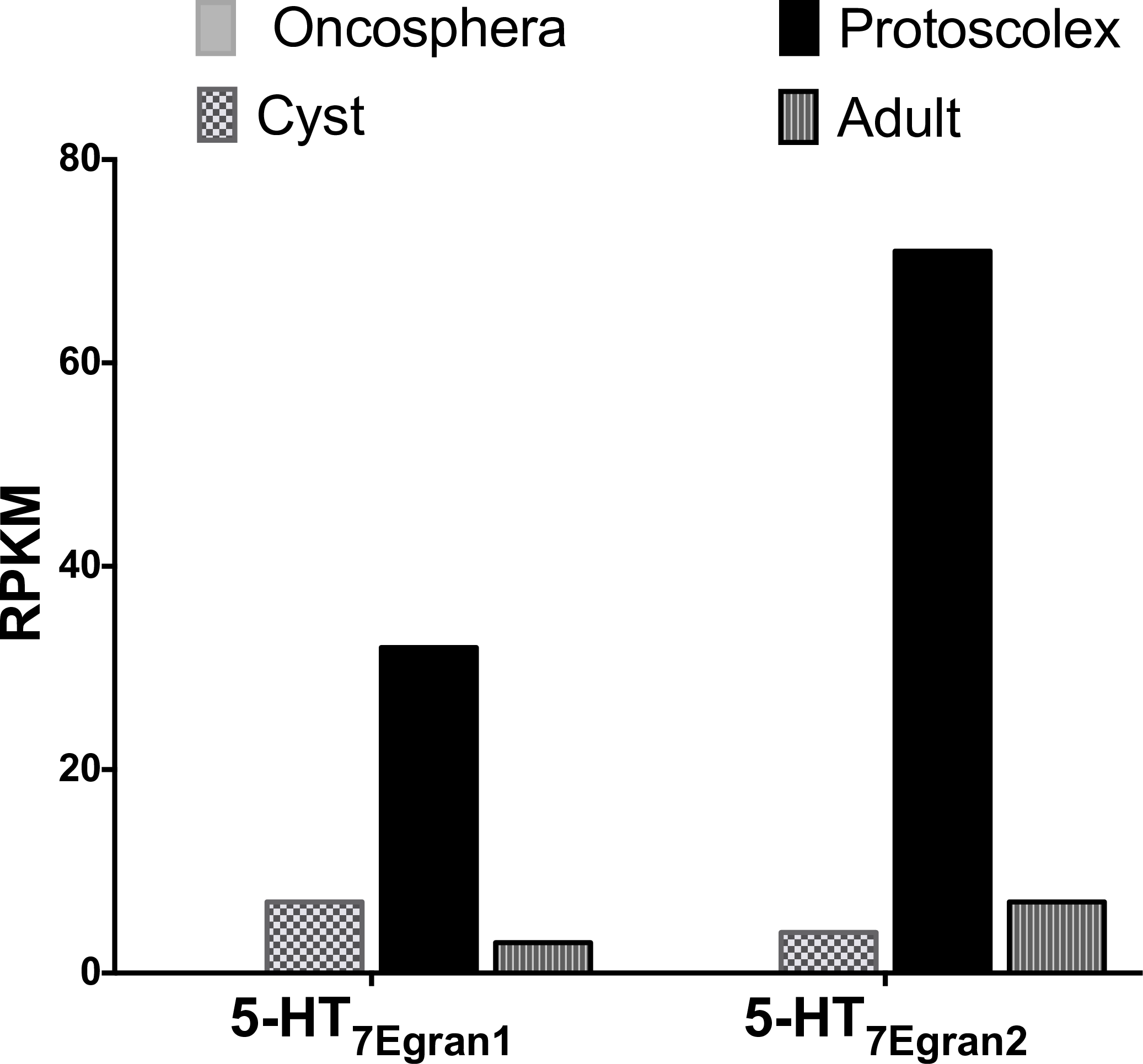

Supplement: S5 Fig — Levels of expression (in RPKM) of 5-HT7Egran1 and 5-HT7Egran2 serotoninergic GPCRs in the oncosphere, cyst, protoscolex and adult stage of Echinococcus granulosus. (TIF) [file pntd.0006267.s007.tif]

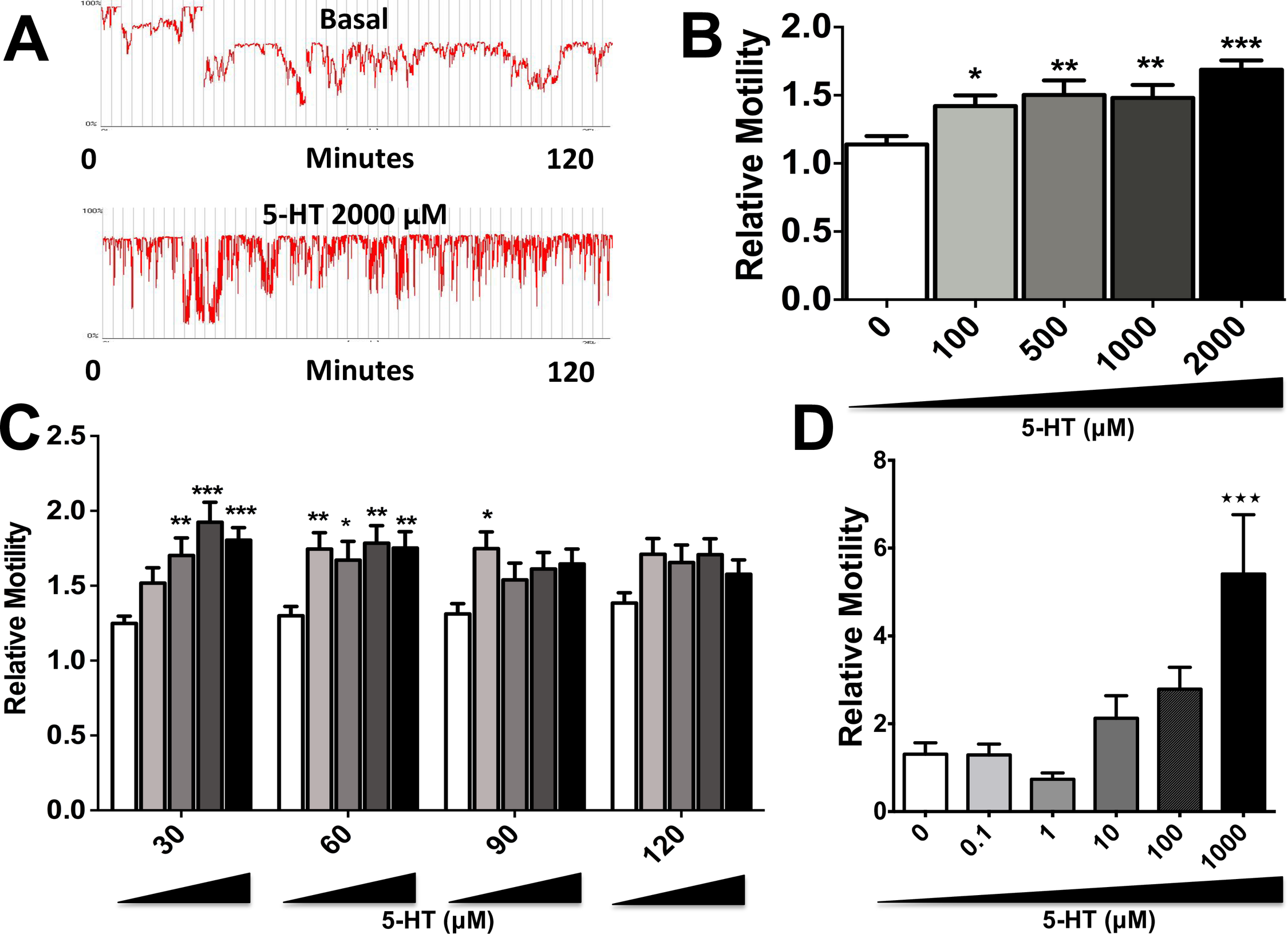

Supplement: S6 Fig — A) Channel activity traces from the WMicrotracker device before (Basal) and after the addition of 2000 μM of serotonin (5-HT 2000 μM) during a period of two hours. B) Relative motility counts without (0 μM, white bar) or after the addition of increasing concentrations of serotonin during a period of two hours. C) Relative motility counts after the addition of the same concentrations of serotonin as in B), but accumulated during a period of 30 minutes and during the intervals of 0–30 (30), 30–60 (60), 60–90 (90) and 90–120 (120) minutes after the addition of 5-HT. D) Relative motility without (0 μM, white bar) or after the addition of increasing concentrations of serotonin during a period of 30 minutes by video imaging. ANOVA and Dunnet post comparison tests were performed. Asterisks indicate treatments found to be significantly different from the controls (*P ≤ 0.05, **P ≤ 0.01 and ***P ≤ 0.001). Most of the experiments were performed using 16 technical replicates per treatment condition and repeated with four different biological samples with the exception of the experiment showed in D, in which eight technical replicates and repeated with three different biological samples. (TIF) [file pntd.0006267.s008.tif]
